# Development and validation of a potency assay matrix for optimized and consistent manufacture of clinical mesenchymal stem/stromal cells

**DOI:** 10.3389/fimmu.2026.1725191

**Published:** 2026-02-19

**Authors:** Patrick Niekamp, Dongsheng Gu, Jie Jiang, Erik J. Woods, Brian H. Johnstone

**Affiliations:** Ossium Health, Inc., Indianapolis, IN, United States

**Keywords:** conditioned media (CM), extracellular vesicles (EVs), IDO - indoleamine 2 3-dioxygenase, M2-macrophages, macrophage polarization, mesenchymal stem cell, potency assay/release criteria, regulatory T (Treg) cells

## Abstract

**Introduction:**

Mesenchymal stem/stromal cells (MSCs) are being evaluated as cell-based therapies for inflammatory and immune-mediated disorders. However, variability in clinical efficacy and a lack of validated potency assays have impeded regulatory approval for commercialization. Here, we report on our success with developing a matrix of potency assays for evaluating the therapeutic fitness of bone marrow-derived MSCs and demonstrate that the cells consistently suppress T cell proliferation, induce regulatory T cell differentiation, and polarize monocytes into anti-inflammatory M2 macrophages.

**Methods:**

Vertebrae were recovered from consented and screened organ donors by Organ Procurement Organizations and shipped on ice to a central processing facility for isolation of vertebral body bone marrow. MSCs were cultured in a xenogeneic-free medium and characterized based on established markers and expanded for 4 passages. Modulation of immune cells isolated from peripheral blood was evaluated using T cell suppression assays, macrophage polarization, regulatory T cell (Treg) induction and monocyte/macrophage chemoattraction assays.

**Results:**

Mechanistic studies revealed that potency is mediated by MSC-secreted immunoregulatory molecules, including macrophage colony-stimulating factor (M-CSF), transforming growth factor-β1 (TGFβ1), and the chemokine CCL2, as well as by tryptophan depletion via the cytoplasmic protein indoleamine 3,4 dioxygenase-1 (IDO1). Additionally, we show that MSCs secrete high levels of extracellular vesicles which potently induce an anti-inflammatory phenotype in T cells and monocytes. These findings were employed to develop a matrix of surrogate potency assays which consistently demonstrated predicted *in vitro* functionality of MSCs derived from 10 donors.

**Discussion:**

This potency assay platform provides a critical tool for ensuring the quality and consistency of MSC products and will facilitate clinical translation by demonstrating comparability between MSC donors as well as manufactured lots and potentially predicting therapeutic efficacy in clinical trials.

## Introduction

Mesenchymal stem/stromal cells (MSCs) are multipotent progenitor cells capable of differentiating into a variety of cell types, including osteoblasts, chondrocytes, and adipocytes (1). Originally identified in the bone marrow, MSCs have since been isolated from a wide range of tissues such as adipose tissue, umbilical cord, and dental pulp (2, 3). Beyond their regenerative potential, MSCs possess robust immunomodulatory properties, influencing both innate and adaptive immune responses through the secretion of cytokines, growth factors, and by direct cell-cell interactions (4). These characteristics have positioned MSCs as promising therapeutic agents in a variety of inflammatory and immune-mediated disorders. While the plethora of immunoregulatory properties possessed by MSCs suggests therapeutic potential as a cellular therapy, it is important to validate the exact mechanism of action in each disease context.

A landmark for MSC therapies occurred in 2004 with the clinical demonstration that bone marrow-derived MSCs (BM-MSCs) were effective in treating steroid-refractory acute Graft-versus-Host Disease (SR-aGvHD) (5). Over the last two decades, the immunomodulatory and tissue repair properties of MSCs have been explored therapeutically in a range of diseases characterized by extreme immune system dysregulation causing pathological tissue damage (6, 7). Multiple early phase clinical trials with small numbers of patients have demonstrated a strong safety profile and favorable response rates for a variety of indications (8, 9). However, larger studies have produced mixed results (10). The variation in outcomes is likely driven by multiple factors, including differences in MSC expansion protocols, cell dose, patient populations, donor and source tissue variability, and whether the cells were cryopreserved or freshly cultured at the time of administration (11–16).

The general lack of predictive disease biomarkers and disease-relevant potency assays remain major obstacles, underscoring the need for further research into the mechanism driving clinical responses. Furthermore, the U.S. Food and Drug Administration (FDA) requires the implementation of validated potency assays for cell-based therapies to demonstrate biological activity and ensure consistent manufacturing quality (13, 14). These assays should measure cellular functions related to the mechanism of action for addressing the intended clinical indication and incorporate orthogonal assays to measure different dimensions of potency. The lack of clear and correlative potency assays has plagued previous late-stage trials - most notably Mesoblast’s SR-aGVHD trial with remestemcel-L (17). These issues were eventually resolved, but at the expense of much time and, presumably, money, with FDA clearance in December 2024 of a biological license application for remestemcel-L for treatment of SR-aGVHD in pediatric patients (18).

We are developing BM-MSCs derived from deceased organ donor vertebral body bone marrow as a cellular therapy for inflammatory diseases (19). This BM-MSC product has been optimized for high post-thaw viability through cell cycle synchronization prior to cryopreservation which prevents lethal DNA damage (20). Furthermore, immediate post-thaw immunomodulatory function is enhanced by priming the cells with interferon-γ (IFNγ) prior to cryopreservation (21). To facilitate clinical development, initially for GvHD, immunomodulatory properties were first evaluated and then assays that are compatible with a Good Manufacturing Practices (GMP)-compliant manufacturing and testing environment were developed.

In this study, we demonstrated that bone marrow-derived MSCs from deceased donors robustly suppress T cell proliferation, promote regulatory T cell (Treg) differentiation, and induce anti-inflammatory M2 macrophage polarization. These effects were mediated through multiple distinct mechanisms. We further identified and validated surrogate potency assays based on these immunomodulatory mechanisms, establishing a quantitative matrix to evaluate lot-to-lot consistency. This matrix provides a foundation for correlating *in vitro* potency with clinical outcomes in future trials.

## Materials and methods

### Cells and cell culture

Deceased donor-derived vertebral bone marrow MSCs were isolated, cultured and expanded utilizing our published methods (19). Individual MSC donor characteristics are presented in **Supplementary Table 1**. Conformance to the International Society of Cellular Therapy (ISCT) standards for MSC characterization (22) (e.g., surface marker expression, colony forming capacity and trilineage differentiation) was previously established (19) and presented again here for the two main donor MSCs (identified as 046 and 063) used in the present studies (**Supplementary Figure 1**). Unless stated otherwise, experiments were performed with MSCs derived from donor 063.

MSC-CM was produced by culturing freshly thawed MSCs for 3 days in RPMI/10%FBS at a seeding cell density of 450x10^3^ cells per well of a 6-well plate (VWR, 820050-844). To remove cells and cell debris, the supernatant was spun at 2,000g for 10 mins. Peripheral blood mononuclear cells (PBMCs) were isolated from buffy coats (Versiti Blood Center, Indianapolis, IN, USA) using Ficoll-Paque^®^ (Cytiva, Marlborough, MA, USA) density gradient centrifugation. To account for biological variability, peripheral blood from a total of 22 donors was collected for isolation of PBMCs used in this study (**Supplementary Table 1**). T cells were isolated from PBMCs using the EasySep Human T Cell Isolation Kit (SCT, 17951) according to manufacturer’s instructions. Monocytes were isolated from PBMCs using the EasySep Human Monocyte Isolation Kit (SCT, 19359) according to manufacturer’s instructions. CD4^+^ T cells were isolated using the EasySep Human CD4+ T cells isolation kit (SCT, 17952). PBMCs and T cells were labeled with carboxyfluorescein succinimidyl ester (CFSE; BD Bioscience) at a concentration of 5µM per 20x10^6^ cells for PBMCs and 2.5µM per 20x10^6^ cells for T cells. Cells were incubated for 15mins at 37 °C, washed twice with access RPMI/10%FBS, followed by cryopreservation in 90% FBS/10% Me_2_SO.

Spleens from C57BL/6 mice were mechanically dissociated through a 40 μm pore size nylon cell strainer to obtain single-cell suspensions. Cells were labeled with CFSE and cryopreserved in freezing medium containing 10% DMSO and 90% FBS for later use.

For co-culture studies, thawed MSCs were seeded in RPMI/10%FBS at the indicated cell densities using either a 96- or 48-well plate (VWR, 734–2327 and 10062-898, respectively). PBMCs were activated using an αCD3ϵ antibody (Bio X Cell, Lebanon, NH, USA) at a concentration of 150 ng/mL and an αCD28 antibody (InVivoMab, BE0248) at a concentration of 1µg/mL (see **supplemental Table 2** for a complete list of antibodies used in this study). Purified T cells were activated using Dynabeads™ Human T-Activator CD3/CD28 for T Cell Expansion and Activation (ThermoFisher, 11161D) at a ratio of one conjugated bead per cell. IL-2 (PeproTech, 200-02-10UG) was added where indicated.

For experiments with purified EVs, purified T cells were plated and stimulated as above and then EVs or cRPMI control at the indicated concentrations were added. For Treg induction experiments, cells were harvested after 3 days and stained for intracellular (FoxP3) or extracellular (CD25 and C127) protein expression as quantified by flow cytometry (see **Supplementary Table 2** for a list of conjugated antibodies). Cell-free supernatants were also collected for ELISA.

For activation of mouse T cells, isolated splenocytes were stimulated with Dynabeads^®^ Mouse T-Activator CD3/CD28 (Gibco, 11452D) at a cell-to-bead ratio of 2:1. A total of 2 × 10^5^ splenocytes per well were cultured in 96-well plates in RPMI-1640 supplemented with 10% FBS and 50 µM 2-mercaptoethanol for 4 days.

Monocytes were cultured by seeding 80x10^3^ cells in RPMI/10%FBS in 96-well plates and differentiated to either M1 macrophages using 50ng/mL GM-CSF (Thermo Fisher, 300-03-50UG) and 100ng/mL IFNγ (R&D Systems, 285-IF-100/CF), or M2 macrophages using 50ng/mL M-CSF (ThermoFisher, PHC9504) and 10ng/mL IL-10 (ThermoFisher, 200-10-50UG) or cultured with 40x10^3^ MSCs or 50% MSC-CM for 3 days using 96-well plates. Where indicated, 2µg/mL M-CSF neutralizing antibody (R&D systems, MAB216-100), 2µg/mL MerTK antibody (R&D Systems, AF891), 50nM CSF1R inhibitor (Selleckchem, S7725) or 2.5µM MerTKi (Selleckchem, S7342) was added.

### T cell suppression assays

MSCs were co-cultured with PBMCs or purified T cells for at 37 °C for 3 or 4 days at the indicated cell densities using either 96- or 48-well plates. Cell proliferation was determined by CSFE dilution by flow cytometry and the replication index was calculated (see Source Data for formula). For IFNγ neutralization, an IFNγ-neutralizing antibody (BioXcell, BE0235, clone B133.5) was added to the co-culture system at various concentrations, as indicated in the Results section. IDO1 inhibitor 1-Methyl-L-tryptophan (Sigma-Aldrich, 447439) were added at a concentration of 500μM and Trp (Sigma-Aldrich, T0254) was supplemented at a concentration of 5mg/L.

### Flow cytometry

Single cell suspensions were stained with the appropriate antibodies (**Supplementary Table 2**) in MACS buffer (0.5% BSA, 2mM EDTA, PBS) for 15–30 mins on ice in the dark. DAPI was added immediately before acquisition. For intracellular staining, cells were stained for viability using Ghost Dye Violet 450 (Cytek, 13-0863-T100) or Fixable Viability Dye eFluo 780 (ThermoFisher, 65-0865-14) for 10 mins on ice in the dark, followed by surface antibody staining for 30mins on ice in the dark. Next, cells were fixed with IC Fixation Buffer (ThermoFisher, 88-8824-00) for 15mins at RT, followed by permeabilization with Perm Buffer (ThermoFisher, 88-8824-00) for 5mins at RT and stained for 1hr with antibodies at RT. A NovoCyte 2060R flow cytometer (Agilent Technologies, Santa Clara, CA, USA) was used for flow cytometry data acquisition. Data was analyzed using NovoExpress software (version 1.5.6).

For intracellular IDO-1 assessment, MSC were fixed and permeabilized using eBioscience™ Intracellular Fixation & Permeabilization Buffer Set (ThermoFisher Scientific) and stained with APC conjugated anti-IDO-1 antibody (clone eyedio; Invitrogen) according to manufacturer’s instruction. Labelled cells were analyzed via flow cytometry.

Flow cytometry data were pre-gated to exclude debris and doublets using forward and side scatter parameters (FSC-H vs SSC-H; FSC-A vs FSC-H; SSC-A vs SSC-H), and non-viable events were excluded using a viability dye where applicable. Compensation and gating were established using single-stained controls and fluorescence-minus-one (FMO) controls for each fluorochrome in combination with appropriate controls (e.g. non-differentiation or non-stimulated cells), as well as isotype controls for intracellular stainings. Data were acquired under identical instrument settings across experiments, with minimum event thresholds applied per sample.

### Enzyme-linked immunosorbent assay

IL-10 concentration in the supernatants was evaluated using the IL-10 ELISA kit (Invitrogen, 88-7106-88) following manufacturer’s instructions. M-CFS concentration in the supernatants was evaluated using the M-CSF (CSF1) ELISA kit (Invitrogen, EHCSF1). CCL2 concentration in the supernatants was evaluated using the MCP-1/CCL2 ELISA kit (Invitrogen, 88-7399-22). CD63 concentration in the supernatants and extracellular vesicles (EVs) was evaluated using the CD63 ELISA kit (Invitrogen, EH95RB). TGFβ1 concentration was evaluated using the TGFβ1 ELISA kit (R&D Systems, DB100C) following manufacturer’s instructions. TGFβ1 was activated by adding 20µL 1M HCl to 200µL pre-diluted samples and an incubation for 1 hour at RT, followed by neutralization with 20µL 1M NaOH. Where indicated, samples were not activated (**Supplementary Figure 7D**). For measurement of M-CSF and TGFβ on EVs were isolated and enriched as described below.

### Transwell migration assay

16hrs before the assay, 300,000 MSCs were seeded per well of a 24-well plate (Corning, 3398, 3µm pore size) without the insert in 500µL cRPMI or 500µL MSC-CM was added per well without the insert. 1hr before the addition of monocytes 2µg/mL anti-CCL2 (R&D Systems, MAB679) or 75ng/mL recombinant CCL2 (ThermoFisher, 300-04) were added where indicated. Insert was carefully inserted to the well plates and 120,000 monocytes were added to the insert in 200µL cRPMI. A control of untreated cells was included. After 1hr, the insert was carefully removed, the cells in the well plate were vigorously resuspended, followed by rinsing of the well plate and processed via flow cytometry to determine the number of migrated monocytes. To determine polarization, the lower well cells (migrated cells) were placed back into the incubator. The monocytes attached to the insert (none-migrated cells) were vigorously resuspended, and the transferred into a new well of a 24-well plate and cultured in 500µl cRPMI. After 48hrs, the cells were harvested and processed via flow cytometry to determine monocyte polarization.

### Isolation and quantification of EVs

MSCs were cultured at a seeding cell density of 4.5x10^5^ cells per well of a 6-well plate for 72hrs. The supernatant was carefully harvested and centrifuged at 1,000g for 10mins to remove cells and cell debris. 6mL of cleared supernatant was loaded onto a Vivaspin 6 Centrifugal Concentrator with a pore size of 300,000 MW cut-off (Vivaproducts, VS0652) and centrifuged for 50mins at 4,500g at RT. A sample from the flow-through was taken and 3mL PBS were added to the top to wash EVs and the sample was centrifuged again at 4,500g for 30mins at RT. EVs were resuspended to a final volume of 400µL in RPMI/10%FBS and stored at -80 °C until further processing. EV numbers were determined using the ZetaView (Particle Metrix) and a CD63 ELISA (ThermoFisher, EH95RB). Acquisition settings or the ZetaView were sensitivity: 85; shutter speed: 70; frame rate: 30 fps. Additionally, for flow cytometry staining, EVs were incubated with CD63 capture beads (Abcam #ab239686, clone TEA3/18) according to manufacturer’s instructions in PBS at RT overnight, followed by staining of EVs for PE/Cy7 anti-human CD63 (BioLegend #353010, clone H5C6) and FITC anti-human CD73 (BioLegend 344016, clone AD2).

EVs were further characterized by Western blot for the presence and absence of discrete proteins according to recommendations MISEV2023 (23). EVs were analyzed by Western blot to confirm protein marker expression. EV and MSC lysates were prepared in 4× Laemmli buffer containing 100× protease inhibitors under non-reducing conditions and heated at 70 °C for 10 min. Samples of equal amount of total protein (15ug) were separated by 4–20% Mini-PROTEAN^®^ TGX™ Precast Protein Gels (Invitrogen, 4561093) using Invitrogen 10× Tris/Glycine/SDS running buffer (1610732) and transferred to PVDF membranes (BioRad, 1620177). Membranes were blocked with 3% BSA in TBS-T, incubated with primary antibodies against positive EV markers (CD9, CD81, ALIX, TSG101) and negative markers (GRP94, Calnexin, TOM20) at 1:1000 dilution, followed by HRP-conjugated secondary antibody (Goat anti Rabbit IgG HRP Conjugate, Boster Biological Tech, BA1054-0.5) 1:5000. Primary Antibodies used were from Cell Signaling: CD9 (D8O1A), CD81 (D3N2D) (56039S), TSG101 (E6V1X), Alix (E6P9B) 80S, Calnexin (2433S), Grp94 (2104S), and Tom20 (D8T4N). Bands were visualized by SuperSignal West Pico PLUS Chemiluminescent Substrate, ThermoFisher, 34577).

### RNA isolation and bulk RNA sequencing

For sequencing of monocytes, freshly thawed cells were cultured at a concentration of 10^6^ cells per well of a 6-well plate in 2mL for 24hrs. Monocytes were either cultured with 50% MSC-CM, 50ng/mL GM-CSF plus 100ng/mL IFNγ, 50ng/mL M-CSF plus 10ng/mL IL-10, or left untreated.

RNA was isolated using the RNeasy kit (Qiagen, 74104) according to manufacturer’s instructions. Isolated RNA was quantified using the NanoDrop8000 spectrometer. Samples were processed by poly-A-enrichment-based library preparation for sequencing in a NovaSeq PE150 Flowcell performed by NovoGene (Sacramento, CA, USA).

The aligned FPKM reads were loaded into R. Differentially expressed genes were identified using the DESeq2 package. Gene ontology analysis was performed using the clusterProfiler package. The full codes are deposited on GitHub (https://github.com/pniekamp113/Niekamp_Ossium-Health)

### cDNA generation and quantification via qPCR

cDNA was generated using High-capacity cDNA Reverse Transcription Kit (Applied Biosystems by ThermoFisher, 4368814) according to the manufacturer’s instructions. The reverse transcription was performed in BioRad T100 Thermal Cycler. The generated cDNA was used for quantification PCR procedure, with TaqMan Universal PCR Master Mix (Applied Biosystems by ThermoFisher,4304437) and qPCR primers, in Mic PCR machine (Bio Molecular Systems). The cycling conditions were as follow: 50 °C for 2 min followed by 95 °C for 10 min, 40 cycles of 95 °C for 15 sec and 60 °C for 60 sec, with a final hold 72 °C for 60 sec. Primers were obtained from ThermoFisher (IL10: Hs00961622 m1 IL10, TNF: Hs00174128 m1 TNF, IL1B: Hs01555410 m1 IL1B). GAPDH (Applied Biosystems, 4332649) was used as housekeeping gene to calculate fold change in gene expression.

### Cellular interaction analysis

A publicly available scRNA seq dataset (GSE253355) (24) of the human bone marrow niche enriched for MSCs was used to analyze cellular interaction networks. We employed the CellChat package to analyze cellular interactions using the CellChatDB database (25). Singe cell RNA-seq data was mapped onto a protein-protein interaction network. Significant cell-cell interactions were identified using the default settings, incorporating population size adjustments and calculating mean gene expression using the “trimean” method. The full code is deposited on GitHub (https://github.com/pniekamp113/Niekamp_Ossium-Health).

### CBO-labeled EV uptake assay

Freshly thawed MSCs were stained with CBO (Biotium, 30022) for 20mins at 37 °C in PBS by adding 5µL of CBO per mL PBS at a cell density of 10^6^ cells/mL. Cells were washed twice with access RPMI/10%FBS. MSCs were cultured for 3 days in RPMI/10%FBS at 37 °C at a seeding cell density of 4.5x10^5^ cells per well of a 6-well plate to generate CBO-labeled EVs. EVs were isolated as described above. Freshly thawed monocytes were rested for 1hr at 37 °C in RPMI/10%FBS. EVs were added to 300x10^3^ monocytes and the monocytes were incubated with EVs for 6, 24, and 48hrs. At the indicates time points, the monocytes were harvested and washed twice with PBS. For detection using imaging flow cytometry, the cells were stained with APC anti-CD14 (BioLegend, 398706) and acquired using the Cytek Amnis FlowSight Imaging Flow Cytometer (Cytek Bioscience). For detection via conventional flow cytometry, the cells were stained with the following conjugated antibodies and stain (Biolegend) APC/Cy7 anti-CD14 (M5E2), APC anti-MERTK (590H11G1E3), PE/Cy7 anti-CD163 (GHI/61), and BV785 anti-CD80 (2D10) and DAPI (422801) and acquired using the NovoSight flow cytometer.

### Phagocytosis assay

Monocytes were primed with MSC-derived EVs for 2 days in 96-well plates at a density of 8x10^4^ cells per well. As controls, monocytes were differentiated to M1 or M2 macrophages as described above, or monocytes were incubated with EVs isolated from cRPMI. 3x10^4^ microspheres (Polysciences, 19519-500) were incubated with IgG-FITC (MilliporeSigma, F9636) at a concentration of 5mg/mL overnight at RT at 300rpm shaking. Beads were washed 3x with access PBS to remove unbound IgG-FITC. A small aliquot was checked for labeling efficiency (>90%) and beads were added to monocytes at a multiplicity of infection (MOI) of 2.5 (2x10^5^ beads added per well to 8x10^4^ seeded monocytes). Plates were centrifuged at 1000g for 5mins to synchronize phagocytosis and then incubated at 37 °C for 1hr. Cells were detached on ice with 5mM EDTA for 15mins, washed and stained with CD45-APC (BioLegend, 304012) for 15mins followed by acquisition with the FlowSight Imaging Flow Cytometer.

### Potency matrix

To generate a potency factor matrix, the average values of IDO1, M-CSF, CD63, and CCL2 for 8 donors were determined. The coefficient of variation was calculated by dividing the standard deviation for each factor through the mean. The potency matrix was generated by first calculating the quartiles for each factor and then assigning 0 to 3 points for each factor depending on the quartile placement for each factor. For the weighted potency matrix, the scores for IDO1 and CD63 were multiplied by 2 and the scores for CCL2 were divided by 2.

### Statistical analysis

Statistical analyses, as indicated, were performed using GraphPad Prism statistical analysis software package (version 10.6.1).

### Data and code availability

Software and source code created for this study are publicly available and can be obtained at GitHub at https://github.com/pniekamp113/Niekamp_Ossium-Health. Bulk and single cell RNA-seq data can be downloaded from the NCBI Gene Expression Omnibus (GEO) database with the accession numbers GSE000001 and GSE000002. Public single cell RNA-seq data can be downloaded from the NCBI GEO database with the accession number GSE253355.

## Results

### MSCs possess multiple immunosuppressive properties

**Figure 1:**
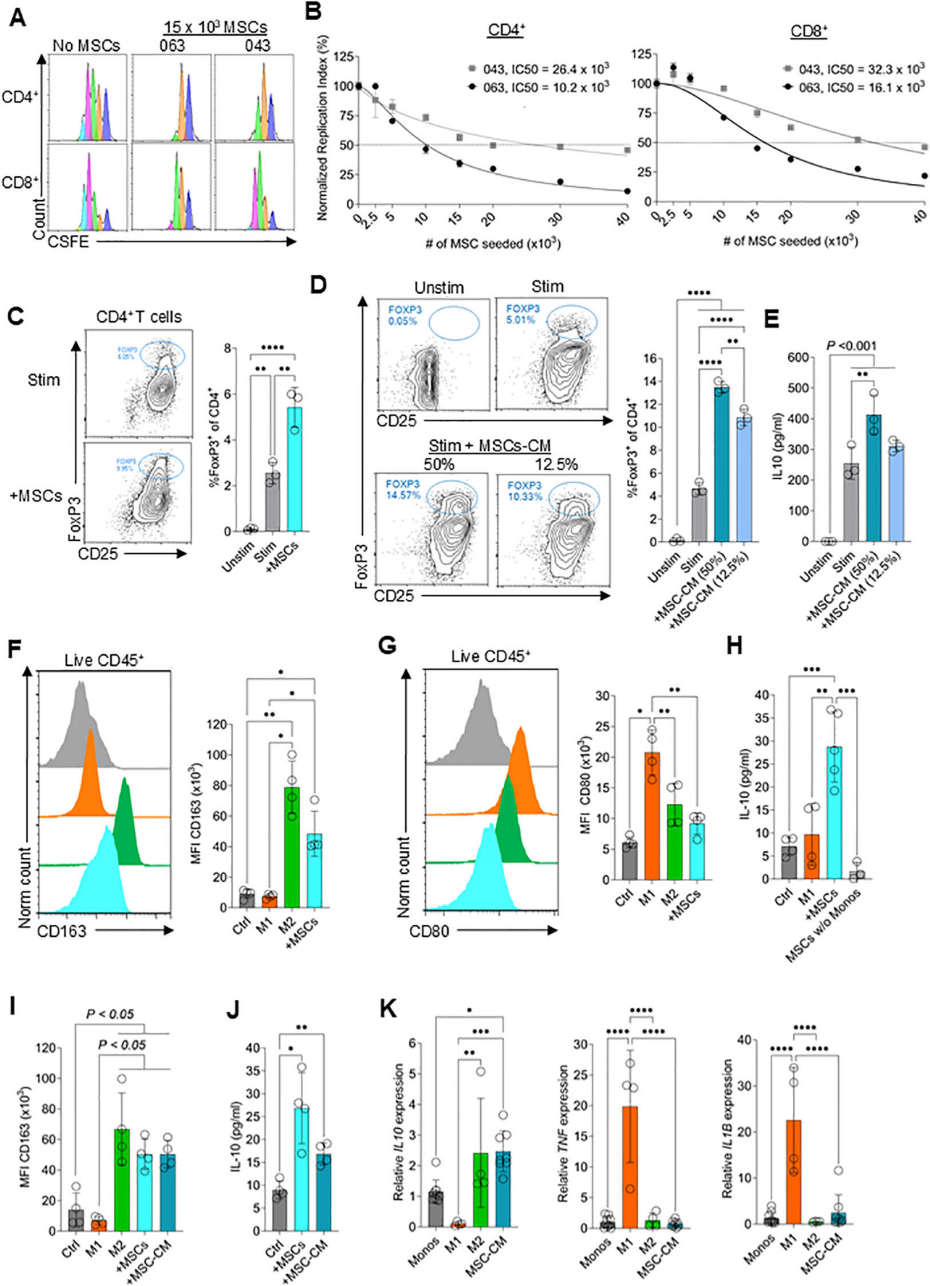
Mesenchymal stem cells inhibit effector T cell proliferation and promote Treg and M2 macrophage polarization. **(A)** Representative flow cytometry plots showing dilution of CFSE in CD4^+^ and CD8^+^ T cells during co-culture of MSCs with peripheral blood mononuclear cells (PBMCs) for 4 days. **(B)** Normalized replication index for CD4^+^ and CD8^+^ T cells with increasing cell numbers of MSCs for MSC donor 063 (black dots) and 043 (grey squares). Data are means ± SD. n = 3 technical replicates using one representative PMBC donor. The number of MSCs that inhibited proliferation by 50% (IC50) was determined using a nonlinear fit model **(C)** Flow cytometry data showing the relative frequency of FOXP3^+^CD25^+^ Tregs among CD4^+^ T cells stimulated with αCD3/αCD28 beads + IL-2 and co-cultured with MSCs (+MSCs), without MSCs (Stim) or cultured without αCD3/αCD28 beads (Unstim) for 4 days. Data are means ± SD. n = 3 technical replicates using one PBMC donor. **(D)** Flow cytometry data showing the frequency of FOXP3^+^ CD25^+^ CD4^+^ T cells stimulated with αCD3/αCD28 beads + IL-2 and cultured with 50% or 12.5% MSC-CM, without MSC-CM (Stim) or cultured without αCD3/αCD28 beads (Unstim) for 4 days. Data are means ± SD. n = 3 technical replicates using one PBMC donor. **(E)** IL-10 levels in the supernatant of T cells stimulated with αCD3/αCD28 beads + IL-2 and cultured with 50% or 12.5% MSC-CM, without MSC-CM (Stim) or cultured without αCD3/αCD28 beads (Unstim) for 3 days. Data are means ± SD. n = 3 technical replicates using one PBMC donor. **(F, G)** Flow cytometry data showing the expression levels of CD163 **(F)** and CD80 **(G)** in monocytes differentiated to M1 macrophages, M2 macrophages, co-cultured with MSCs, or left untreated for 3 days. Data are means ± SD. n = 4 independent replicates using four different PBMC donors. Monocytes were differentiated to M1 macrophages with 50ng/mL GM-CSF and 100ng/mL IFNγ and differentiated to M2 macrophages using 50ng/mL M-CSF and 10ng/mL IL-10. **(H)** IL-10 levels in the supernatant of monocytes differentiated to M1 macrophages, co-cultured with MSCs or left untreated and IL-10 levels in MSCs cultured without monocytes for 3 days. Data are means ± SD. n ≥ 4 independent replicates using three different PBMC donors. **(I)** Flow cytometry data showing the expression levels of CD163 monocytes differentiated to M1 macrophages, M2 macrophages, cultured with 50% MSC-CM, or left untreated for 3 days. Data are means ± SD. n = 4 independent replicates using four different PBMC donors. **(J)** IL-10 levels in the supernatant of monocytes co-cultured with MSCs, cultured with 50% MSC-CM or left untreated for 3 days. Data are means ± SD. n = 4 independent replicates using four different PBMC donors. **(K)** Relative RNA expression levels of *IL10, TNF*, and *IL1B* in monocytes differentiated to M1 macrophages, M2 macrophages, cultured with 50% MSC-CM, or left untreated for 24hrs. Data are means ± SD. n = 4 independent replicates using ≥ 3 different PMBC donors. Statistical significance was determined using one-way ANOVA with Tukey’s HSD test **(C–K)**. *P ≤ 0.05; **P ≤ 0.01; ***P ≤ 0.001; ****P ≤ 0.0001.

*In vitro* proliferation assays are a standard method for assessing the T cell inhibitory function by cells of interest (26). Previous studies demonstrated that inter-donor variability as well as differences in culture conditions can influence the suppressive potential of MSCs (27, 28). To initially evaluate the variation in T cell suppression activity between donors, MSCs derived from two different donors (donors identified as 063 and 043) were co-cultured at different cell densities with CFSE-labeled PBMCs for 4 days (**Figures 1A, B**). MSCs dose-dependently suppressed activated CD4^+^ and CD8^+^ T cell proliferation, with a clear difference in the potency between MSC isolated from the two different donors. The concentration of MSCs required to inhibit T cell proliferation by 50% (i.e., IC50) was 2.6-fold lower for CD4^+^ T cells and 2-fold lower for CD8^+^ T cells with donor 063 MSCs compared to 043 (**Figures 1A, B**). Suppression was independent of other cells in the PBMCs as demonstrated by a strong inhibition of activated purified T cell proliferation, suggesting that MSCs directly suppress T cell proliferation (**Supplementary Figures 2A, B**).

Previous work suggests that MSCs induce CD4^+^ T cell differentiation to Tregs (29, 30). In line with this, we observed that co-culture of MSCs with T cells induced a higher percentage of FoxP3^+^ CD25^+^ CD4^+^ T cells compared to T cells cultured alone (**Figure 1C**). It is unclear if MSCs stimulate Treg differentiation via a cell contact-dependent mechanism, a cell contact-independent mechanism, or both (31). To test this, conditioned media (CM) were generated and added at two different concentrations (50% and 12.5%) to T cells plated in complete (c)RPMI medium. Both CM concentrations induced higher percentages of FoxP3^+^CD25^+^ CD4+ Tregs as well as increased interleukin-10 (IL-10) secretion compared to the control (cRPMI) condition (**Figures 1D, E**). These results demonstrate that MSCs secrete substances that induce FoxP3^+^ Treg formation.

MSCs have been reported to drive monocyte polarization towards anti-inflammatory M2 macrophages (32–34). To validate this in our system, we cultured isolated CD14^+^ monocytes with MSCs and used established M1 and M2 macrophages polarization conditions as control (35–40). Co-culture with MSCs for 3 days selectively polarized purified monocytes to anti-inflammatory CD163^+^ M2 macrophages (**Figure 1F**) with comparatively fewer pro-inflammatory CD80^+^ M1 macrophages present (**Figure 1G**) (41). Additionally, co-culture induced IL-10 expression (**Figure 1H**). IL-10 was not expressed by MSCs alone in the conditions used here. To test if M2 polarization is cell contact-dependent, MSC-CM was used as described above and found to be sufficient to recapitulate the co-culture phenotype (**Figures 1I, J**). Further analysis of cytokine gene expression confirmed that MSC-CM selectively polarized monocytes to anti-inflammatory M2 macrophages without induction of pro-inflammatory M1 macrophages (**Figure 1K**).

Overall, these results confirmed that the deceased donor vertebral bone marrow-MSCs used in this study potently suppress T cell proliferation, induce Treg differentiation, and promote M2 macrophage polarization (i.e. early skewing of monocytes towards a M2 macrophage phenotype). Validating these important immunomodulatory functions establishes the therapeutic potential of this abundant source of MSCs for treating inflammatory diseases. The next steps in development for clinical testing, as described below, involved developing a matrix of potency assays based on each of these functions to monitor the quality of GMP-compliant manufactured cells.

### Development of a robust T cell suppression surrogate assay

T cell suppression is a well described MSC immunomodulatory function (21, 42, 43). Attempts have been made to adapt this assay to a standard which is compatible with regulatory requirements for comparing clinical lots to ensure consistent potency. However, the assay is difficult to standardize, primarily due to the variability in responses between stimulated PBMCs isolated from different donors (13, 44). An alternative would be to measure a surrogate function of the MSCs which strongly correlates with T cell suppression activity. The first described and best characterized mediator of T cell suppression *in vitro* is the IFNγ-inducible cytoplasmic protein IDO1 (21, 42). IDO1 is the first, rate-limiting enzyme metabolizing tryptophan (Trp) in the kynurenine (Kyn) pathway (45). T cells require Trp for proliferation and previous work showed that macrophages and cancer cells deplete Trp in the tumor microenvironment via cell-associated IDO1 activity to inhibit T cell proliferation (46, 47). A positive correlation between IDO1 expression by primed MSCs isolated from different donors and activated T cell suppression has been noted; therefore, levels of IDO1 expression by IFNγ-primed MSCs could be a useful surrogate assay for activated T cell suppression activity (43).

**Figure 2:**
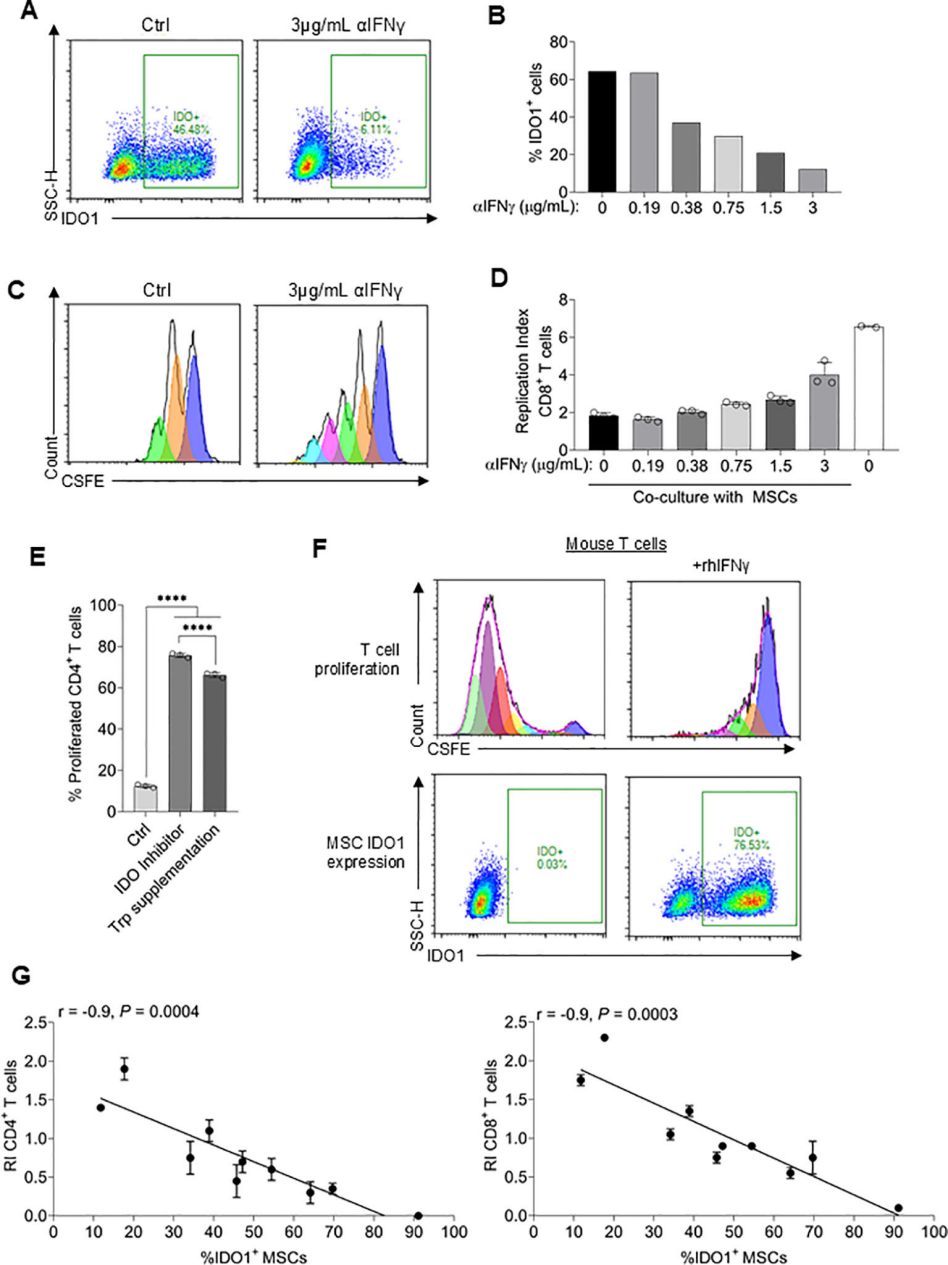
Development of a robust T cell suppression surrogate assay. **(A)** Representative flow plots of intracellular IDO1 expression in MSCs co-cultured with T cells ± 3µg/mL αIFNγ. **(B)** Quantification of IDO1-positive MSCs co-cultured with T cells plus varying concentrations of αIFNγ antibody. The experiment was replicated once using the same PMBC donor and yielded similar results. **(C)** Representative flow cytometry plots of CFSE-dilution in CD8^+^ T cells co-cultured with MSCs ± 3µg/mL αIFNγ. **(D)** Replication index of CD8^+^ T cells co-cultured with MSCs plus varying concentrations of αIFNγ antibody. n ≥ 2 technical replicates using one PBMC donor. MSC donor 063 was used for all experiments. **(E)** Percentage of proliferated CD4^+^ T cells co-cultured with MSCs alone or additionally supplemented with an IDO1 inhibitor or tryptophan. n = 3 technical replicates using one PBMC donor. **(F)** T cells isolated from mouse spleen were co-cultured with MSCs ± recombinant human IFNγ. Representative CFSE-dilution of CD4^+^ T cells (top) and IDO1 expression in MSCs is shown (bottom). The experiment was repeated once, yielding similar results **(G)** Correlation of the replication index of CD4^+^ and CD8^+^ T cells and %IDO1+ MSCs using 10 different MSC donors. n = 2 technical replicates using one PBMC donor. Statistical significance was determined using one-way ANOVA with Tukey’s HSD test **(E)**. Pearson r was calculated, and the statistical significance was determined using a two-tailed *t*-test. ****P ≤ 0.0001.

As a first step to developing a robust surrogate assay, IDO1 expression was confirmed to be the dominate mediator of T cell suppression in the *in vitro* assay conditions used. An IFNγ neutralizing antibody (αIFNγ) was used to block IDO1 expression, reducing IDO1 expression from ~60% to <10% at the highest antibody concentration (**Figures 2A, B**). The degree of T cell suppression was correspondingly reduced by increasing concentrations of αIFNγ (**Figures 2C, D**). Neutralizing IFNγ without MSC co-culture had no effect on the proliferation of T cells (data not shown). Additionally, an IDO1 inhibitor as well as re-supplementing the medium with Trp was sufficient to rescue T cell proliferation (**Figure 2E**). To further demonstrate the dependence of T cell suppression on IFNγ-induced IDO1 expression, we took advantage of the inability of murine IFNγ to cross-react with human IFNγ receptor (48). Intracellular IDO1 expression was undetectable and murine T cell proliferation was unaffected when human MSCs were co-cultured with mouse splenocytes (**Figure 2F**). However, adding recombinant human IFNγ to the co-culture induced IDO1 expression in MSCs and significantly suppressed mouse T cell proliferation (**Figure 2F**).

The above data confirmed that MSC IDO1 expression is obligately dependent on IFNγ priming in a dose-dependent manner and, therefore, suggested that measurement of IDO1 expression could be a facile surrogate assay for T cell suppression activity. To test this, MSCs derived from 10 different donors were assayed for IFNγ-induced IDO1 expression and activated T cell suppression activity. A strong negative correlation (r = -0.9; P<0.001) was observed between IDO1 expression (range:12% to 91%) and CD4 as well as CD8 T cell proliferation (**Figure 2G**). Overall, these results demonstrate that assaying for IDO1 expression is a valid surrogate for MSC-mediated suppression of activated T cell proliferation and, as such, is a suitable assay for measurement of potency.

### MSCs drive M2 macrophage polarization through secretion of macrophage-colony stimulating factor

**Figure 3:**
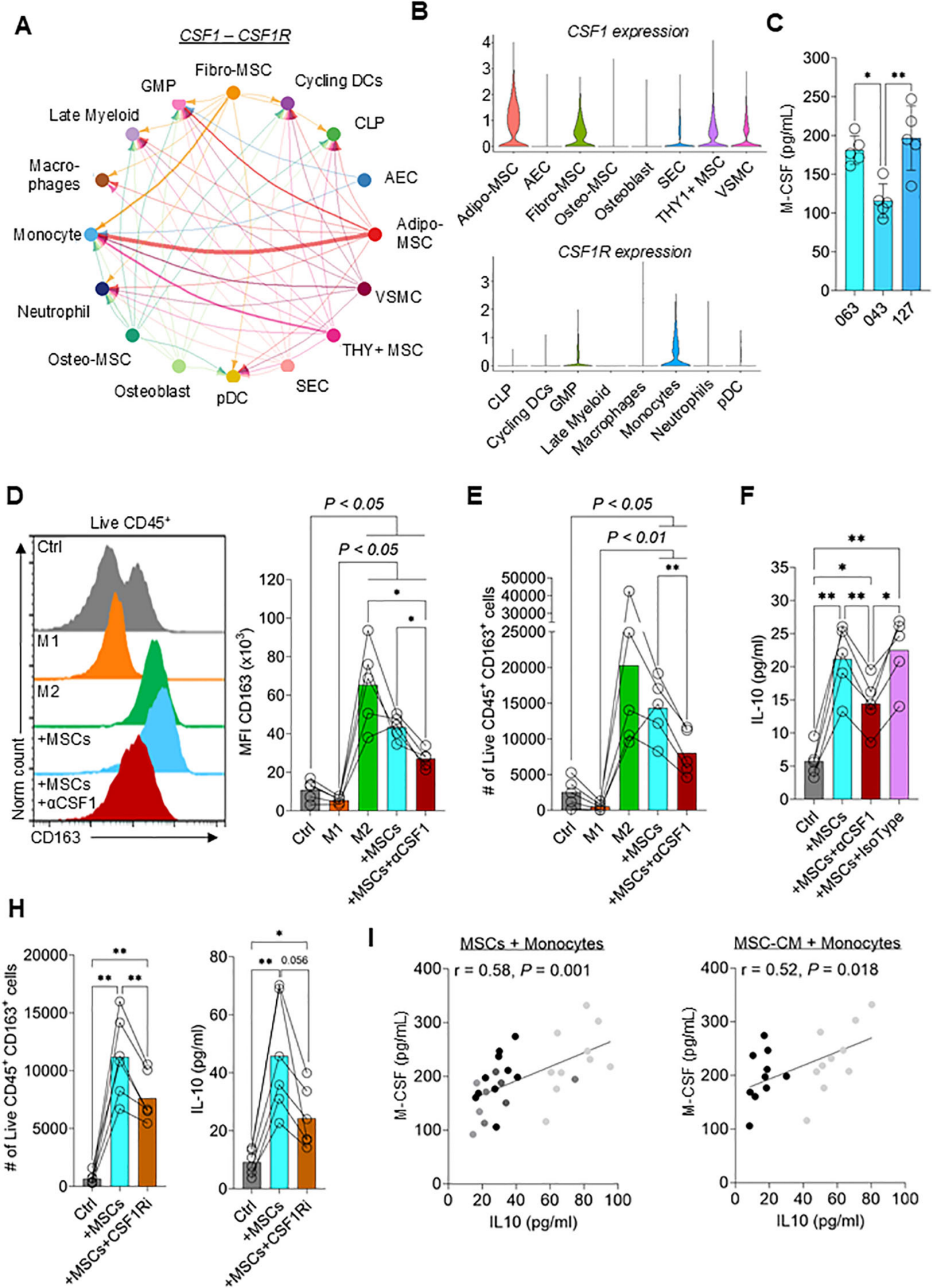
M-CSF secreted by MSCs drives M2 macrophage polarization. **(A)** Chord diagram showing *CSF1 – CSF1R* interaction from publicly available scRNA-seq data set (GSE253355). The thickness of the line corresponds to the strength of the predicted interaction. GMP = granulocyte monocyte progenitor; CLP = common lymphoid progenitor; AEC = arterial endothelial cell; VSMC = vascular smooth muscle cell; SEC = sinusoidal endothelial cell **(B)** Violin plots showing the relative expression of *CSF1* in stromal cell populations (top) and *CSF1R* in myeloid populations (bottom) **(C)** M-CSF levels in the supernatant of three different MSC donors. Data are means ± SD. n = 5 independent replicates using four different PBMC donors. **(D, E)** Flow cytometry data showing **(D)** the mean fluorescence intensity (MFI) of CD163 and **(E)** the number of live CD45^+^ CD163^+^ monocytes differentiated to M1 macrophages, M2 macrophages, co-cultured with MSCs ± neutralizing anti-M-CSF (αM-CSF) antibody or left untreated for 3 days. Data are means ± SD. n = 5 independent replicates using five different PBMC donors. **(F)** IL-10 concentration in the supernatant of monocytes co-cultured with MSCs ± neutralizing anti-M-CSF antibody or isotype control or left untreated for 3 days. Data are means ± SD. n = 5 independent replicates using five different PBMC donors. **(H)** Number of live CD45^+^ CD163^+^ cells and IL-10 concentrations of monocytes co-cultured with MSCs ± CSF1R inhibitor or left untreated for 3 days. Data are means ± SD. n = 6 independent replicates using three different PBMC donors. **(I)** Correlation of IL-10 secreted by monocytes and M-CSF secreted by MSCs using 10 different MSC donors. n ≥ 2 independent replicates with ≥ 2 different PBMC donors were performed. Dot shading corresponds to monocytes derived from each donor. Pearson r was calculated, and the statistical significance was determined using a two-tailed *t*-test. * = p < 0.05, ** = p ≤ 0.01

To understand how MSCs drive M2 polarization, publicly available scRNA seq data of the human bone marrow niche, including stromal cells, was used (24). The authors identified five different MSC populations in the bone marrow. We employed the CellChat package (25) to systematically interrogate interactions between stromal cells and immune cell populations. One of the strongest predicted interactions between MSCs and monocytes is *CSF1* (the gene encoding M-CSF) signaling (**Figure 3A**). *CSF1* is expressed by multiple MSC subsets, including Adipo-MSCs, Fibro-MSCs and THY1^+^ MSCs, while the cognate receptor (*CSF1R*) is expressed at high levels by monocytes (**Figure 3B**). The expression of M-CSF was verified in the supernatants of cultured MSCs obtained from three different donors (**Figure 3C**). To test if MSC-secreted M-CSF induces M2 polarization, an M-CSF neutralizing antibody was used to inactivate receptor binding. Neutralization of M-CSF significantly reduced MSC-induced upregulation of the M2 macrophage-specific marker CD163 (**Figure 3D**). Additionally, M-CSF neutralization reduced live CD45^+^ CD163^+^ cell numbers and IL-10 expression (**Figures 3E, F**). In line with this observation, blocking *CSF1R* signaling using a CSF1R inhibitor also significantly reduced the live CD45^+^ CD163^+^ cell numbers and IL-10 expression (**Figure 3H**). To test the suitability of measuring M-CSF as a surrogate for M2 macrophage polarization, MSCs isolated from 10 different donors, or CM derived from each, were separately added to monocyte/macrophage cultures. A significant correlation was observed (*P* < 0.001 and *P* < 0.05 when either MSC or CM, respectively, was used) between M-CSF levels in the supernatant and IL-10 released by monocytes/macrophages (**Figure 3I**). These data suggest that measuring M-CSF release is an additional surrogate assay for MSC immunomodulatory potency.

### CCL2 secretion by MSCs facilitates macrophage polarization through recruitment of monocytes

Neutralizing M-CSF significantly reduced M2 polarization but did not completely abolish it, suggesting that other factors support polarization. Bulk RNA-seq of monocytes cultured for 24hrs with MSC-CM was employed to identify candidate factors driving M2 polarization (**Supplementary Figure 3A**). Controls were untreated monocytes (Ctrl) and monocytes polarized to either M1 or M2 macrophages by the addition of recombinant cytokines. M1 macrophages demonstrated the most extreme differences in gene expression profile compared to the three other groups (**Supplementary Figure 3B**). To further understand the similarities and differences between Ctrl, MSC-CM and M2 samples, we excluded M1 macrophages (**Supplementary Figure 3C**). This analysis revealed multiple gene clusters that were regulated by MSC-CM. For instance, genes in group (i) that were decreased in M2 macrophages compared to Ctrl and MSC-CM include *HLA-DMA*, *HLA-DMB*, *HLA-DPB*, and *HLA-DRA*, which are involved in antigen presentation (**Supplementary Figure 3C**). Genes in group (ii) that were increased in MSC-CM-induced macrophages and M2 macrophages controls compared to the monocyte control include *CD163, IL10*, and *MERTK*. Each of the proteins expressed from these transcripts are implicated in anti-inflammatory processes (49–51).

**Figure 4:**
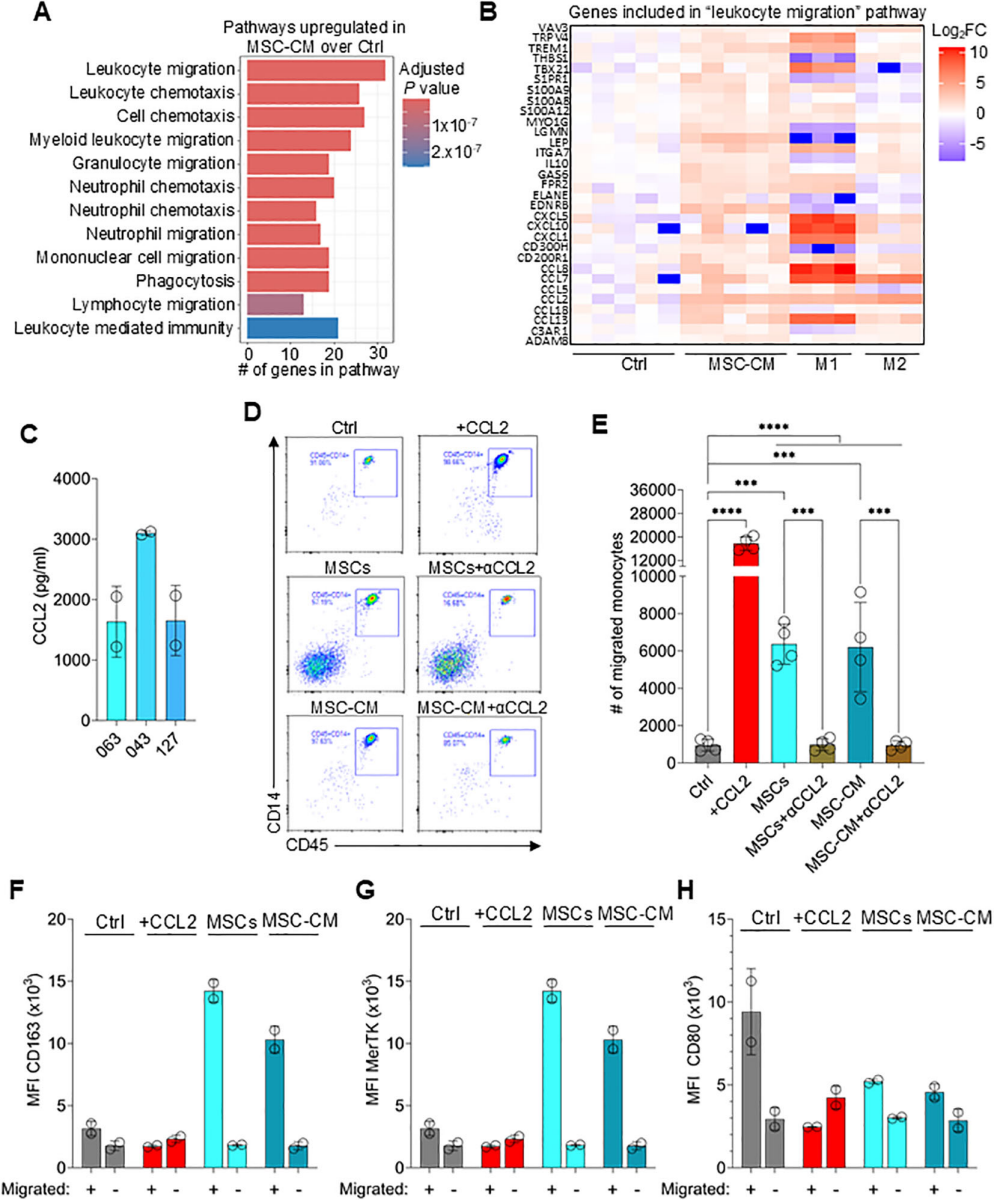
MSCs secrete CCL2 necessary for monocyte recruitment. **(A)** Gene Ontology (GO) biological pathway (BP) analysis showing the top 12 pathways enriched in MSC-CM-treated monocytes over untreated control (Ctrl) samples. **(B)** Heatmap showing the log_2_ fold-change of genes included in the “leukocyte migration” pathway between Ctrl, MSC-CM, M1, and M2 macrophages. **(C)** CCL2 levels in the supernatant obtained from cultured MSCs derived from 3 different donors. Data are means ± SD. n = 2 independent replicates using two PBMC donors. **(D)** Representative flow plots of lower chamber monocytes as in **(E)**. **(E)** Number of migrated monocytes to lower chamber at 1hr incubation. Data are means ± SD. n = 4 independent replicates using four PBMC donors. **(F-H)** MFI of CD163, MerTK and CD80 on monocytes migrated (lower chamber) or not migrated (upper chamber) and cultured for 3 days. Data are means ± SD. n = 2 independent replicates using two PBMC donors. Statistical significance was determined using one-way ANOVA with Tukey’s HSD test **(D)**. ***P ≤ 0.001; ****P ≤ 0.0001.

To further understand how MSC-CM affects monocytes/macrophages polarization to M2 macrophages, genes upregulated in CM-treated monocytes compared to the control were compared and evaluated using gene ontology (GO) analysis (**Figure 4A**). Pathways enriched in CM-treated monocytes included “leukocyte migration” and “cell chemotaxis” (**Figures 4A, B**; full list of genes and pathways can be found in Source Data file). MSCs express relatively high levels of CCL2, a potent chemokine necessary for monocyte recruitment (**Figure 4C**) (52, 53). To test if MSC-secreted CCL2 drives monocyte migration, we performed a transwell experiment, whereby MSCs or CM were added to the bottom well and migration of monocytes added to the top well was tracked. Both, MSCs and CM effectively stimulated monocyte migration from the upper chamber to the lower chamber, which was abolished by CCL2 neutralization (**Figures 4D, E**).

To determine if migration towards MSCs is required for effective M2 macrophage polarization, the migration experiment was repeated followed by culturing migrated (lower chamber) and non-migrated (upper chamber) monocytes for an additional 3 days. Monocytes that migrated towards MSCs or CM followed by 3-day culture expressed high levels of CD163 and MerTK but relatively low levels of CD80, confirming M2 polarization (**Figures 4F–H**). However, non-migrated monocytes or monocytes migrated via establishing a recombinant CCL2 gradient did not upregulate CD163 or MerTK expression. These results indicate that CCL2 secreted by MSCs recruit monocytes which are then converted to M2 macrophages via localized high concentrations of MSC-derived molecules.

### MSC potency is mediated through secretion of EVs

**Figure 5:**
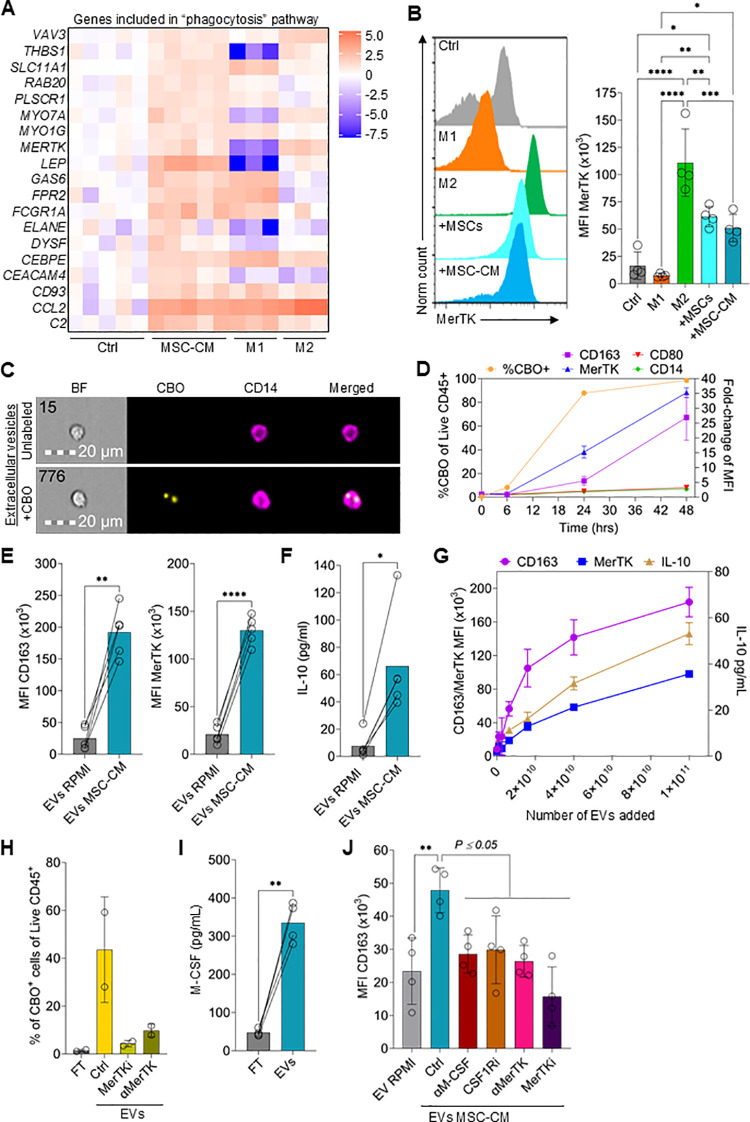
MSCs produce high levels of EVs that drive M2 polarization. **(A)** Heatmap showing the log_2_ fold-change in expression of genes included in the “phagocytosis” pathway between Ctrl, MSC-CM, M1, and M2 macrophages. **(B)** Flow cytometry data showing the expression levels of MerTK on monocytes differentiated to M1 macrophages, M2 macrophages, co-cultured with MSCs, cultured with 50% MSC-CM, or left untreated for 3 days. Data are means ± SD. n = 4 independent replicates using four different PBMC donors. **(C)** Representative images of monocytes cultured with EVs derived from CBO-labeled MSCs for 24hrs. **(D)** Time course of CBO-labeled EV uptake and expression of CD163, MerTK, CD80, and CD14 on monocytes. Data are means ± SD. n = 2 technical replicates using one PBMC donors. **(E)** CD163 and MerTK expression on monocytes treated with EVs isolated from MSC-CM (EVs MSC-CM) or cRPMI (EVs RPMI) for 3 days. Data are means ± SD. n = 4 independent EV preparation from one MSC donor (donor 063), using four different PBMC donors. **(F)** IL-10 levels in the supernatant of monocytes treated with EV isolated from MSC-CM or cRPMI for 3 days. Data are means ± SD. n = 4 independent EV preparation from one MSC donor (donor 063) and using four different PBMC donors. **(G)** CD163 and MerTK expression and IL-10 levels at different concentrations of EVs. **(H)** Percentage of CBO^+^ monocytes of monocytes incubated with isolated CBO labeled EVs ± a MerTK inhibitor or αMerTK antibody or incubated with the EV isolation column flowthrough (FT). Data are means ± SD. n = 2 independent experiments using two different PBMC donors. **(I)** M-CSF ELISA levels of EV FT or EVs from MSCs. Data are means ± SD. n = 4 independent experiments using four different PBMC donors. **(J)** CD163 MFI of monocytes incubated with EVs isolated from cRPMI, MSC-EVs (Ctrl) or MSC-EVs plus an αM-CSF antibody, CSF1Ri, an αMerTK antibody, or MerTKi. Data are means ± SD. n = 4 independent experiments using four different PBMC donors. Statistical significance was determined using one-way ANOVA with Tukey’s HSD test. *P ≤ 0.05; **P ≤ 0.01; ***P ≤ 0.001; ****P ≤ 0.0001.

Interestingly, GO pathways analysis indicated that cell-free MSC-CM enriched phagocytosis in monocytes/macrophages (**Figure 4A**). Upregulated genes in the phagocytosis pathway included *MERTK* and *GAS6*, which mediate efferocytosis and uptake of extracellular vesicles (EVs) *(***Figure 5A**) (54). We first confirmed increased MerTK protein levels in monocytes/macrophages co-cultured with MSCs or cultured with CM (**Figure 5B**). Next, EVs from CM were isolated using columns with a molecular cut-off of 300 kDa, followed by characterization and quantification of the EVs using nanoparticle tracking analysis (NTA), CD63 ELISAs, and CD63 capture beads (**Supplementary Figures 4A–F**). The EVs were positive for the markers CD9, ALIX, TSG101 and CD81; while negative for GRP94 and Calnexin (**Supplementary Figure 4A**) (23). The average EV size was ~80nm, with the majority of particles ranging between 30 and 150nm in size, which are classically defined as exosomes 3(**Supplementary Figures 4B, C**) (55). On average, ~5.2x10^11^ EVs/mL were present in CM and there was a clear correlation (r=0.86; P<0.001) between CD63 concentration measured by ELISA and EV numbers determined with NTA (**Supplementary Figures 4D, E**).

To test if MSC EVs are taken up by monocytes, MSCs were labeled with the cytoplasmic membrane dye CellBrite Orange (CBO) (56), the supernatant was harvested, and EVs were isolated. Uptake of labeled EVs was assessed by both imaging and conventional flow cytometric analysis of monocytes (**Figures 5C, D**). After 6hrs, <10% of monocytes had a detectable CBO-signal (**Figure 5D**). However, after 24hrs and 48hrs the percentage of CBO-positive monocytes increased to 87% and 98%, respectively. Interestingly, EV uptake correlated with an early increase in MerTK expression followed by CD163 upregulation (**Figure 5D**). As expected, CD80 expression remained low.

Using four different EV preparations and four different monocyte donors, strong M2 macrophage polarization of monocytes by MSC-derived EVs was demonstrated, as shown by increased expression of CD163, MerTK and IL-10 after a 3-day culture (**Figures 5E, F**). There was also a clear concentration-dependent relationship between EV numbers added to monocytes and CD163, MerTK and IL-10 induction (**Figure 5G**). Additionally, when comparing CBO-positive and CBO-negative monocytes from the same sample, CBO uptake was clearly correlated (r=0.86; *P* < 0.001) with CD163 expression (**Supplementary Figures 5A, B**). As MerTK expression by monocytes was rapidly upregulated by EVs and previous research suggests a role of MerTK in the uptake of EVs (54, 57), we reasoned that blocking MerTK would reduce CBO-labeled EV uptake. Both a MerTK neutralizing antibody or a MerTK inhibitor clearly reduced the uptake of CBO-labeled EVs (**Figure 5H**). A recent publication indicated that M-CSF is expressed on EVs secreted by cancer cells (58). Indeed, M-CSF was detected in both EVs and the flow-through obtained during column purification and, moreover, was highly enriched in purified EV preparations (**Figure 5I**). Of note, EV isolation resulted in a 15-fold concentration of EVs (start volume vs. final EV volume). This process likely contributed to the high levels of M-CSF measured on EVs compared to the FT or compared to CM (**Figure 3C**).

To demonstrate the direct involvement of MerTK-mediated uptake of EVs, leading to M2 polarization by EV-associated M-CSF, both pathways were inhibited. Blocking MerTK activity on monocytes/macrophages using either a neutralizing antibody or small molecule inhibitor reduced CD163 expression (**Figure 5J**). Similarly, blocking either EV M-CSF activity or monocyte/macrophage CSF1R activity also reduced CD163 expression (**Figure 5J**).

Finally, expression of genes increased in the “phagocytosis” pathway included those involved in the conventional phagocytosis pathway, such as *THBS1, CD93, FCGR1A*, and *VAV3* (**Figure 4A**) (59–61). Phagocytic clearing of autoreactive T cells has also been implemented in the anti-inflammatory function of M2 macrophages in diseases such as GvHD (62). Therefore, we tested if priming monocytes with EVs increased the phagocytic potential of monocytes/macrophages. M2 macrophages and monocytes primed with MSC-derived EVs phagocytized IgG-opsonized microbeads more effectively compared to M1 macrophages or untreated monocytes (**Supplementary Figures 6A–C**).

Overall, these data suggest that MSCs induce M2 macrophage polarization through EVs displaying M-CSF which activates CSF1R either preceding or following endocytosis through the MerTK/GAS6 pathway with the net result of increased anti-inflammatory activity.

### MSC EVs drive Treg polarization

**Figure 6:**
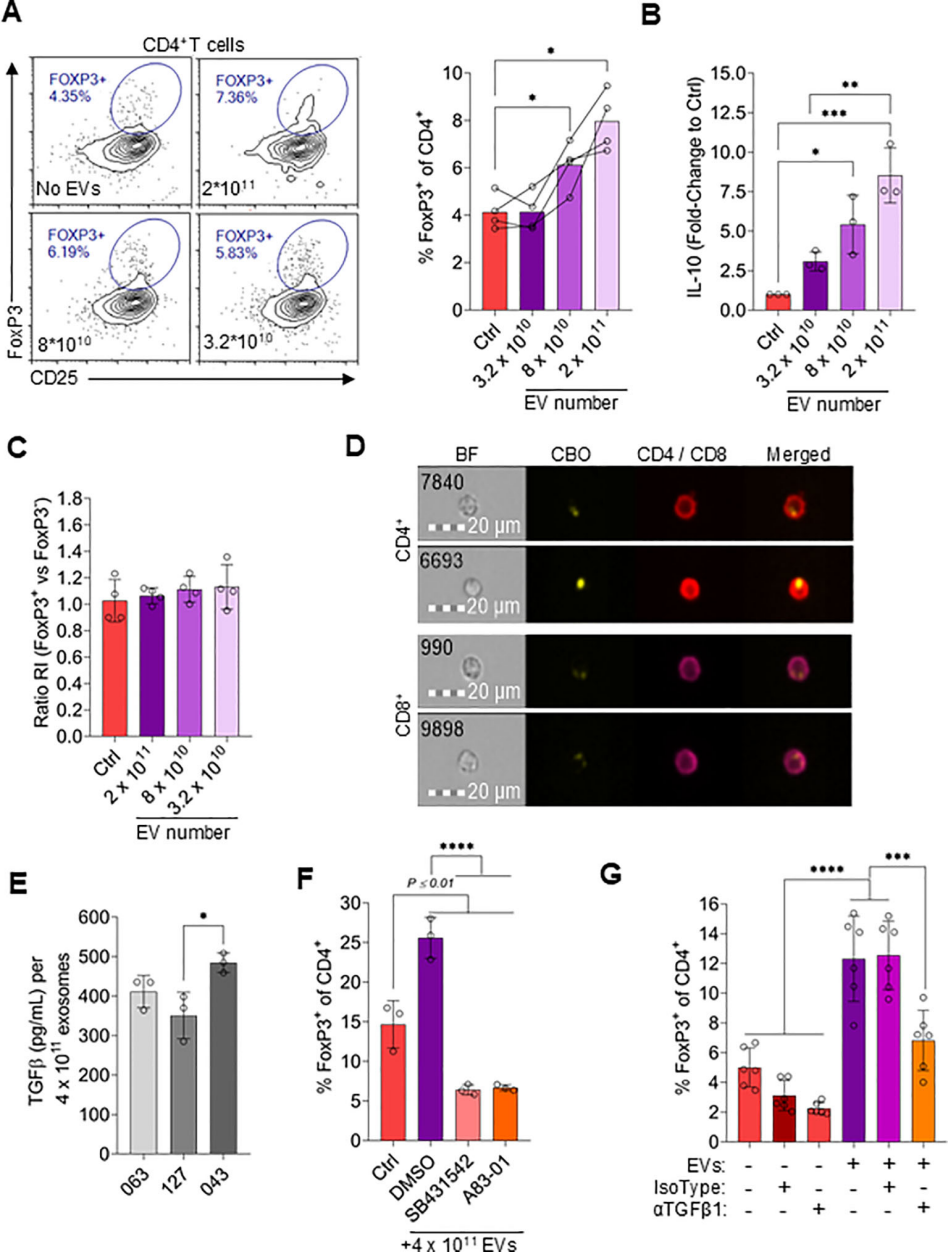
MSC-derived EVs drive Treg polarization. **(A)** Representative flow cytometry plots and quantification of FoxP3 and CD25 expression in CD4^+^ T cells treated with different numbers of EVs or without EVs (Ctrl). Data are means ± SD. n = 4 technical replicates using one PBMC donors. **(B)** IL-10 levels in the supernatant of T cells incubating with a range of EV numbers or without EVs. Data are means ± SD. n = 3 technical replicates using one PBMC donors. **(C)** Replication index ratio of FoxP3-positive vs. FoxP3-negative CD4^+^ T cells treated with different numbers of EVs or without EVs. Data are means ± SD. n = 3 technical replicates using one PBMC donors. **(D)** Representative imaging flow cytometry images of CD4^+^ and CD8^+^ T cells incubated with CBO-labeled EVs for 24hrs. **(E)** ELISA TGFβ1 levels on EVs (4 x 10^11^ EVs used for ELISA) from MSC-CM derived from three different MSC donors. Data are means ± SD. n = 3 technical replicates using three independent EVs preparations per MSC donor. **(F)** Frequency of FoxP3^+^ cells among CD4^+^ T cells treated with 4 x 10^11^ EVs for 3 days in combination with 5µM SB431542 or 1µM A83–01 or vehicle control (DMSO) or incubated without EVs (Ctrl). Data are means ± SD. n = 3 technical replicates using one PBMC donors. **(G)** Frequency of FoxP3^+^ cells among CD4^+^ T cells treated with or without 4 x 10^11^ EVs for 3 days ± 5µg/mL anti-TGFβ1 antibody or isotype control. Data are means ± SD. n ≥ 3 technical replicates using ≥ 3 different PMBC donors. Statistical significance was determined using one-way ANOVA with Tukey’s HSD test. *P ≤ 0.05; **P ≤ 0.01; ***P ≤ 0.001; ****P ≤ 0.0001.

Considering that MSC-CM induces Treg polarization (**Figure 1D**), we speculated that EVs may also contribute to Treg induction. To test this, CD4^+^ T cells were incubated with different concentrations of EVs (**Figure 6A**). Indeed, there was an EV concentration-dependent increase in FoxP3 expression, with approximately twice as many CD4^+^ cells expressing FoxP3 when incubated with 2x10^11^ EVs compared to without EVs (**Figure 6A**). Similarly, IL-10 levels in the culture supernatant increased proportionally to EV concentration (**Figure 6B**). To understand if EVs released by MSCs promoted *de novo* Treg differentiation or preferentially supported Treg proliferation, the replication index of FoxP3^+^ and FoxP3^-^ CD4^+^ cells was compared (**Figure 6C**). Both CD4^+^ T cell populations replicated to the same extent in the presence or absence of EVs, suggesting that EVs did not preferentially stimulate Treg proliferation.

Using imaging flow cytometry we further observed that EVs labeled with CBO were taken up by CD4^+^ and CD8^+^ T cells (**Figure 6D**). To identify specific components that induce Treg formation, EVs were first assayed for TGFβ1, a well described induction factor for Treg differentiation [61]. Relatively high levels of TGFβ1 were detected on EVs (**Figure 6E**). Interestingly, the TGFβ1 concentration on EVs varied between EVs derived from MSC isolated from different donors (**Figure 6E**). To confirm that EV-associated TGFβ1 was functional and directly induced Treg formation, its activity was blocked using two different TGFβR1 pathway inhibitors (63, 64), resulting in significantly reduced EV-mediated Treg induction (**Figure 6F**). Additionally, a neutralizing antibody was used as a complementary method to confirm EV-mediated Treg induction via associated TGFβ1 (**Figure 6G**). Of note, FoxP3 expression in the control sample varied significantly based on the specific donor T cells used, which is a well-documented phenomenon (65, 66). It was confirmed that TFGβ1 associated with EVs was necessary for Treg induction and not other potential sources by reisolating EVs to remove free antibody before testing (**Supplementary Figures 7A, B**).

The Latency-Associated-Peptide (LAP) noncovalently binds TGFβ1, thereby preventing activity 37659098. We speculated that neutralizing LAP function could increase active TGFβ1 concentrations. Indeed, a 50% increase in Treg abundance was observed when EVs were incubated for 3hrs with an anti-LAP antibody before addition of EVs to T cells (**Supplementary Figure 7C**). Additionally, neutralization of LAP with an anti-LAP antibody was sufficient to detect active TFGβ1 without the need of activation using HCl (**Supplementary Figure 7D**). Collectively, these data indicate that functional TGFβ1 associated with MSC-derived EVs is able to promote *de novo* Treg induction *in vitro*.

### A quantitative potency assay matrix for qualifying MSCs used in clinical testing

Heterogeneity of potency between manufactured lots has severely impeded commercial development of some MSC therapies (67, 68). To ensure inter-batch consistency of cellular therapies used in clinical trials, the FDA requires potency assays as release criteria [15]. The present study has identified four potency factors (IDO1, M-CSF, EVs, and CCL2) and demonstrated correlations between the quantities of each and the corresponding *in vitro* immunosuppressive activity. Thus, we have developed a matrix of assays that are valid surrogates for measuring different immunomodulatory functions of MSCs.

**Figure 7:**
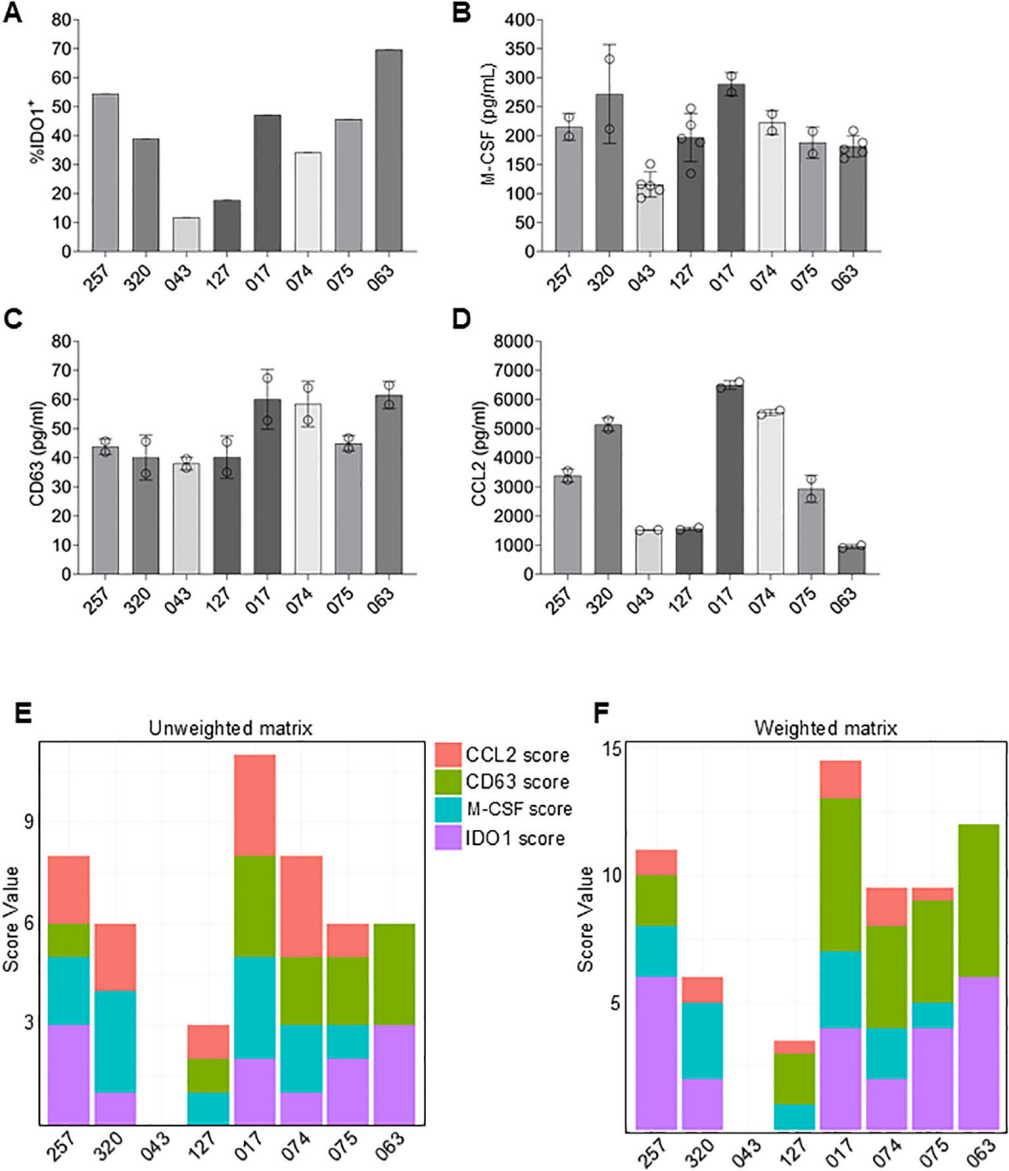
Development of a potency matrix to enhance consistency of MSCs as cell therapy product. **(A–D)** Quantification of intracellular IDO1 expression **(A)**, or secretion of M-CSF **(B)**, CD63 **(C)**, or CCL2 **(D)** by MSCs from 8 different donors. Data are means ± SD. **(E)** Potency matrix whereby 0 to 3 points are assigned to each MSC donor for each potency factor based on quartiles. **(F)** Weighted potency matrix whereby 2 points per quartile are assigned for IDO1 and EV scores and ½ point per quartile for CCL2 scores.

To demonstrate the utility of this assay matrix for assessing MSC potency, MSCs recovered from vertebral body bone marrow obtained from different 8 different donors, were assayed for M-CSF, CD63 (EV concentration) and CCL2 expression as well as percentage of IDO1^+^ cells following IFNγ stimulation (**Figures 7A–D**). For each potency factor, the range between the highest value and lowest value varied with a high coefficient of variation (CV) for IDO1 (47.3%) and CCL2 (60.5%) and smaller CVs for M-CSF (25.8%) and CD63 (20.5%). To evaluate overall potency, we assigned scores to each donor depending on the detected levels of each potency factor (**Figures 7E, F**). For each factor, 0 to 3 points were assigned depending on quartile levels. For instance, donor 320 received a total score of 6 based on the following calculation: IDO1 levels were in the second lowest quartile (1 point), M-CSF levels were in the highest quartile (3 points), CD63 levels were in the lowest quartile (0 points) and CCL2 levels were in the second highest quartile (2 points). Overall analysis of the potency matrix for each of the 8 MSC isolates suggests a high potency of donor 017, followed by donors 257 and 074.

An additional consideration is that not all four potency factors are equal in their impact on immune cell function. For example, activated T cell suppression by IDO1 activity as well as M2 polarization induced by EVs both potently affected immune suppression activity; whereas, CCL2 by itself elicited chemoattraction of monocytes, but, in isolation, did not alter immune cell function. Therefore, each factor was assigned a weighted score to reflect its potential to directly modulate the immune system. Thus, 2X multipliers were added to scores for IDO1, M-CSF and CD63 to account for their relative importance for immune suppression. Conversely, a multiplier of 0.5X was used for CCL2 to account for its relatively lesser importance. The resulting weighted matrix shifts the ranking slightly. MSCs from donor 017 is still predicted to have the highest potency, followed by the MSCs from donor 063 and 257.

These data demonstrate the utility of the developed matrix of surrogate potency assays for screening donor-derived MSCs. By analogy, the matrix is fully expected to similarly provide meaningful comparisons between manufactured lots produced from a single donor MSC to detect changes in the final product. The utility of the potency matrix will be validated before incorporating these assays into testing of GMP manufactured lots. Future planned clinical trials in GvHD patients will evaluate the correspondence between *in vitro* potency and clinical efficacy.

## Discussion

In this study, we characterized multiple immunomodulatory mechanisms employed by MSCs that are in clinical development for GvHD and other inflammatory diseases. In so doing we uncovered the influence on T cell and monocyte functions and phenotypes through secreted factors, metabolic regulation (i.e. Trp depletion), and EV signaling. Our data demonstrate that MSCs suppress effector T cell proliferation, promote Treg induction, and drive monocyte polarization toward an anti-inflammatory M2 macrophage phenotype. The broad applicability of these findings was enhanced by testing immunomodulatory function of a total of 10 MSC isolates from donors age 14-48 (both male and female) using immune cells isolated from a total of 22 male and female donors (ages 18-84). These findings provide further insights into the multiple mechanisms by which MSCs regulate immune cell populations. Moreover, the variability observed in individual potency factor expression between MSCs obtained from different donors may explain the heterogeneity observed in clinical responses to MSC therapies (67, 68). The development of a robust matrix of orthogonal immunomodulatory potency assays allows for multifactorial assessment of *in vitro* activity to better maintain quality control during manufacturing; thus, potentially limiting variations in clinical responses.

The demonstration that IFNγ-stimulated MSCs isolated from deceased donor vertebral body bone marrow suppress T cell proliferation validates previous findings that these cells are no different than iliac crest-derived MSCs obtained from healthy, living volunteer donors (19). These results also corroborate previous reports establishing the importance of IDO1 in MSC-mediated immune regulation and highlight its central role in MSC potency (46, 47). Importantly, a strong correlation was observed between levels of IDO1 expressed by MSCs isolated from different donors and the degree of suppression of T cell proliferation, supporting its use as an indicator of functional potency.

In addition to suppression of T cell activity, it was demonstrated that MSCs orchestrate monocyte polarization toward an M2 phenotype through a coordinated process involving chemoattraction, surface receptor signaling, and EV-mediated communication. Chemoattraction towards MSCs via a CCL2 gradient enhances the polarizing effect of secreted M-CSF, which is reinforced through enhanced MerTK signaling induced by EV uptake. These results build on prior work implicating M-CSF in macrophage polarization (40) and further establish EVs as essential vectors of MSC-mediated immune regulation (69, 70). We also show that MSC-derived EVs carry bioactive M-CSF and that blocking M-CSF or MerTK signaling impairs the acquisition of an M2 phenotype.

The combinatorial effects of MSC-secreted factors provide a coordinated mechanism for affecting immunomodulation within the inflammatory milieu of diseased tissues. Alternatively, given the predominant entrapment of intravenously infused MSCs in lungs, the cells could promote systemic effects (71, 72). A mechanism based on lung-entrapped MSCs promoting circulating macrophage efferocytosis has been proposed to explain therapeutic effects on distal tissues (73). Our findings suggest that MSC-secreted CCL2 could promote recruitment of circulating monocytes to the lungs where they would be polarized by M-CSF and subsequently migrate to lesions in distal tissues. It is also possible that soluble molecules and EVs secreted by MSCs circulate to regulate immune cells in other tissues (71, 74).

In particular, our findings highlight a critical immunoregulatory role of MSC-derived EVs in promoting the induction of Tregs. These EVs were found to carry bioactive TGFβ1, a well-established cytokine that drives FoxP3 expression and Treg lineage commitment (75). The EV-mediated Treg induction was dependent on TGFβ1, as blocking its signaling abrogated Treg formation, and enhancing its activation via LAP neutralization further amplified the response. These data underscore the capacity of MSC-derived EVs.

Together, our findings reveal that MSCs engage multiple, complementary pathways to modulate the immune microenvironment, explaining observations of potent immunomodulation in diverse diseased tissues. From a translational perspective, this work underscores the importance of using a matrix of functionally validated surrogate potency factors that modulate distinct pathways directly relevant to the proposed mechanism of action for treating a specific disease indication. These markers not only reflect the biological activity of MSCs but also serve as potency metrics for manufactured lots that are required by regulatory agencies to ensure quality and consistency of products used in the clinic (76).

Finally, while our study delineates several core mechanisms of immune modulation by MSCs *in vitro*, future clinical studies will demonstrate whether these modulated pathways reflect the actual mechanism of action in the clinical disease scenario. Particularly relevant will be to determine whether the mechanisms described here will be therapeutic within the context of complex inflammatory environments, comorbidities, diverse health histories and demographics of patients. Future studies could also explore how MSC-derived EV cargo composition influences other immune cell subsets and whether MSCs from different tissues show similar functional profiles. Elucidating these context-dependent responses will be essential for the rational design of next-generation cellular therapies.

## Data Availability

The datasets presented in this study can be found in online repositories. The names of the repository/repositories and accession number(s) can be found below:\ https://www.ncbi.nlm.nih.gov/geo/, GSE000001 https://www.ncbi.nlm.nih.gov/geo/, GSE000002.

## Supplementary material

Supplementary Figure 1 Characterization of MSCs. MSCs from donors 043 and 063 were cultured to passage 4 to assess surface marker phenotype, colony forming unit-fibroblast (CFU-F) potential and trilineage differentiation. (A) Means ± SD for expression of each surface marker. (B) Representative flow cytometry histograms. (C) Means ± SD for 14 day CFU-F assays. (D) Representative images of MSCs after adipogenic, osteogenic and chondrogenic differentiation and staining.

Supplementary Figure 2 MSCs suppress proliferation of isolated T cells. **(A)** Representative flow cytometry plots and quantification of CFSE dilution and replication index of CD4^+^ T cells cultured alone (No MSCs) or co-cultured with 20,000 (20k) or 40,000 (40k) MSCs (MSC donor 063). **(B)** Representative flow cytometry plots and quantification of CFSE dilution and replication index of CD8^+^ T cells cultured alone (No MSCs) or co-cultured with 20,000 (20k) or 40,000 (40k) MSCs (MSC donor 063). Data are means ± SD. n ≥ 3 technical replicates using one PBMC donor. Statistical significance was determined using one-way ANOVA with Tukey’s HSD test. ***P* ≤ 0.01; *****P* ≤ 0.0001.

Supplementary Figure 3 Cluster heatmap of monocytes differentiated to M1 or M2 macrophages or treated with MSC-CM. **(A)** Schematic presentation of bulk RNA-seq of monocytes. **(B)** Cluster heatmap showing DEGs with a Log_2_ fold change of ≥ 3. **(C)** Cluster heatmap of DEGs with a Log_2_ fold change of ≥ 1 plus zoom-in showing genes of selected clusters.

Supplementary Figure 4 Quantification of MSC-derived EVs. **(A)** Western blot analysis of EVs and 063 MSC whole cell lysates. Each lane contained 15 µg total protein. Molecular weights were estimated based on MW size markers. (**B**) Size distribution of MSC-derived EVs measured by NTA. **(C)** Average size of EVs. **(D)** Average number of EVs/mL. Data are means ± SD. n = 3 independent MSC-EV preparations. **(E)** Correlation between CD63 levels measured via CD63 ELISA and EV numbers measured by NTA. **(F)** Representative flow images of EVs isolated with CD63 capture beads and stained for CD63 and CD73.

Supplementary Figure 5 MerTK- and M-CSF-signaling mediate EV-driven M2 polarization. (**A)** Representative flow images of live CD45^+^ monocytes incubated with CBO-labeled EVs derived from MSCs or unlabeled EVs. **(B)** Representative flow image of CD163 expression in monocytes treated with CBO-labeled EVs (top) and MFI of CD163 in CBO- vs CBO+ monocytes from the same sample (bottom). Data are means ± SD. n = 4 independent experiments using four different PBMC donors. Statistical significance was determined using a paired *t-*test.

Supplementary Figure 6 Extracellular vesicles released by MSCs increase phagocytic capacity of macrophages. **(A)** Representative imaging flow cytometry images of IgG-FITC opsonized microbeads added to monocytes differentiated to M1 or M2 macrophages, monocytes primed with MSCs-EVs or EVs isolated from cRPMI **(B)** Quantification of IgG-FITC microbeads uptake as described in (A). **(C)** Representative flow images of IgG-FITC-positive monocytes. Data are means ± SD. n = 2 independent experiments using two different PBMC donors.

Supplementary Figure 7 Active TGFβ1 on EVs surface drives Treg differentiation. (**A)** Schematic overview of the experiment. MSC-derived EVs were incubated for 3hrs at 37 °C with an anti-TGFβ1 antibody or isotype control and either added directly to T cells (No Wash) or unbound antibodies were washed away, and EVs were re-isolated (Wash). **(B)** Frequency of FoxP3^+^ among CD4^+^ T cells stimulated with αCD3/αCD28 beads + IL-2 and cultured as described in (A). Data are means ± SD. n = 3 technical replicates using one PBMC donor. **(C)** Frequency of FoxP3^+^ among CD4^+^ T cells stimulated with αCD3/αCD28 beads + IL-2 and culture with EVs, EVs + anti-LAP antibody, EVs + isotype control or without EVs (Ctrl). Data are means ± SD. N = 3 technical replicates using one PBMC donor. **(D)** TGFβ1 concentrations on EVs measured via TGFβ1 ELISA without HCl activation of samples. Additionally, the EVs were incubated with 13 µg/mL anti-LAP antibody or isotype control. Data are means ± SD. n = 3 technical replicates. Statistical significance was determined using a one-way ANOVA with Tukey’s HSD test. **P* ≤ 0.05, ***P* ≤ 0.01.

Supplementary Figure 8 Representative flow cytometry gating strategy. Example of the gating strategies for **(A)** CD4+ and CD8+ CSFE labeled stimulated T cells, **(B)** FoxP3 expression in the CD4+ T cell population, **(C)** IDO expression by MSCs, **(D)** monocytes/macrophages in PBMCs, and **(E)** Determining monocyte migration

## References

[1] Pittenger MF Mackay AM Beck SC Jaiswal RK Douglas R Mosca JD . Multilineage potential of adult human mesenchymal stem cells. Science. (1999) 284:143–7. doi: 10.1126/science.284.5411.143, PMID: 10102814

[2] Orbay H Tobita M Mizuno H . Mesenchymal stem cells isolated from adipose and other tissues: basic biological properties and clinical applications. Stem Cells Int. (2012) 2012:461718. doi: 10.1155/2012/461718, PMID: 22666271 PMC3361347

[3] Ledesma-Martinez E Mendoza-Nunez VM Santiago-Osorio E . Mesenchymal stem cells derived from dental pulp: A review. Stem Cells Int. (2016) 2016:4709572. doi: 10.1155/2016/4709572, PMID: 26779263 PMC4686712

[4] Uccelli A Moretta L Pistoia V . Mesenchymal stem cells in health and disease. Nat Rev Immunol. (2008) 8:726–36. doi: 10.1038/nri2395, PMID: 19172693

[5] Le Blanc K Rasmusson I Sundberg B Gotherstrom C Hassan M Uzunel M . Treatment of severe acute graft-versus-host disease with third party haploidentical mesenchymal stem cells. Lancet. (2004) 363:1439–41. doi: 10.1016/S0140-6736(04)16104-7, PMID: 15121408

[6] Le Blanc K Frassoni F Ball L Locatelli F Roelofs H Lewis I . Mesenchymal stem cells for treatment of steroid-resistant, severe, acute graft-versus-host disease: a phase II study. Lancet. (2008) 371:1579–86. doi: 10.1016/S0140-6736(08)60690-X, PMID: 18468541

[7] Panes J Garcia-Olmo D Van Assche G Colombel JF Reinisch W Baumgart DC . Expanded allogeneic adipose-derived mesenchymal stem cells (Cx601) for complex perianal fistulas in Crohn’s disease: a phase 3 randomised, double-blind controlled trial. Lancet. (2016) 388:1281–90. doi: 10.1016/S0140-6736(16)31203-X, PMID: 27477896

[8] Chen X Wang C Yin J Xu J Wei J Zhang Y . Efficacy of mesenchymal stem cell therapy for steroid-refractory acute graft-versus-host disease following allogeneic hematopoietic stem cell transplantation: A systematic review and meta-analysis. PloS One. (2015) 10:e0136991. doi: 10.1371/journal.pone.0136991, PMID: 26323092 PMC4554731

[9] Keklik M Deveci B Celik S Deniz K Gonen ZB Zararsiz G . Safety and efficacy of mesenchymal stromal cell therapy for multi-drug-resistant acute and late-acute graft-versus-host disease following allogeneic hematopoietic stem cell transplantation. Ann Hematol. (2023) 102:1537–47. doi: 10.1007/s00277-023-05216-3, PMID: 37067556

[10] Kadri N Amu S Iacobaeus E Boberg E Le Blanc K . Current perspectives on mesenchymal stromal cell therapy for graft versus host disease. Cell Mol Immunol. (2023) 20:613–25. doi: 10.1038/s41423-023-01022-z, PMID: 37165014 PMC10229573

[11] Koyama I Nadazdin O Boskovic S Ochiai T Smith RN Sykes M . Depletion of CD8 memory T cells for induction of tolerance of a previously transplanted kidney allograft. Am J Transplant. (2007) 7:1055–61. doi: 10.1111/j.1600-6143.2006.01703.x, PMID: 17286617 PMC3785402

[12] von Bahr L Sundberg B Lonnies L Sander B Karbach H Hagglund H . Long-term complications, immunologic effects, and role of passage for outcome in mesenchymal stromal cell therapy. Biol Blood Marrow Transplant. (2012) 18:557–64. doi: 10.1016/j.bbmt.2011.07.023, PMID: 21820393

[13] Galipeau J Krampera M Barrett J Dazzi F Deans RJ DeBruijn J . International Society for Cellular Therapy perspective on immune functional assays for mesenchymal stromal cells as potency release criterion for advanced phase clinical trials. Cytotherapy. (2016) 18:151–9. doi: 10.1016/j.jcyt.2015.11.008, PMID: 26724220 PMC4745114

[14] de Wolf C van de Bovenkamp M Hoefnagel M . Regulatory perspective on *in vitro* potency assays for human mesenchymal stromal cells used in immunotherapy. Cytotherapy. (2017) 19:784–97. doi: 10.1016/j.jcyt.2017.03.076, PMID: 28457740

[15] Gregoire C Ritacco C Hannon M Seidel L Delens L Belle L . Comparison of mesenchymal stromal cells from different origins for the treatment of graft-vs.-host-disease in a humanized mouse model. Front Immunol. (2019) 10:619. doi: 10.3389/fimmu.2019.00619, PMID: 31001253 PMC6454068

[16] Johnstone BH Woods JR Goebel WS Gu D Lin CH Miller HM . Characterization and function of cryopreserved bone marrow from deceased organ donors: A potential viable alternative graft source. Transplant Cell Ther. (2023) 29:95 e91–10. doi: 10.1016/j.jtct.2022.11.010, PMID: 36402456 PMC9918674

[17] Galipeau J . The mesenchymal stromal cells dilemma--does a negative phase III trial of random donor mesenchymal stromal cells in steroid-resistant graft-versus-host disease represent a death knell or a bump in the road? Cytotherapy. (2013) 15:2–8. doi: 10.1016/j.jcyt.2012.10.002, PMID: 23260081

[18] FDA U . FDA approves remestemcel-L-rknd for steroid-refractory acute graft versus host disease in pediatric patients (2024). Available online at: https://www.fda.gov/drugs/resources-information-approved-drugs/fda-approves-remestemcel-l-rknd-steroid-refractory-acute-graft-versus-host-disease-pediatric (Accessed November 19, 2025).

[19] Johnstone BH Miller HM Beck MR Gu D Thirumala S LaFontaine M . Identification and characterization of a large source of primary mesenchymal stem cells tightly adhered to bone surfaces of human vertebral body marrow cavities. Cytotherapy. (2020) 22:617–28. doi: 10.1016/j.jcyt.2020.07.003, PMID: 32873509 PMC8919862

[20] Johnstone BH Gu D Lin CH Du J Woods EJ . Identification of a fundamental cryoinjury mechanism in MSCs and its mitigation through cell-cycle synchronization prior to freezing. Cryobiology. (2023) 113:104592. doi: 10.1016/j.cryobiol.2023.104592, PMID: 37827209

[21] Krampera M Cosmi L Angeli R Pasini A Liotta F Andreini A . Role for interferon-gamma in the immunomodulatory activity of human bone marrow mesenchymal stem cells. Stem Cells. (2006) 24:386–98. doi: 10.1634/stemcells.2005-0008, PMID: 16123384

[22] Dominici M Le Blanc K Mueller I Slaper-Cortenbach I Marini F Krause D . Minimal criteria for defining multipotent mesenchymal stromal cells. The International Society for Cellular Therapy position statement. Cytotherapy. (2006) 8:315–7. doi: 10.1080/14653240600855905, PMID: 16923606

[23] Welsh JA Goberdhan DCI O’Driscoll L Buzas EI Blenkiron C Bussolati B . Minimal information for studies of extracellular vesicles (MISEV2023): From basic to advanced approaches. J Extracell Vesicles. (2024) 13:e12404. doi: 10.1002/jev2.12404, PMID: 38326288 PMC10850029

[24] Bandyopadhyay S Duffy MP Ahn KJ Sussman JH Pang M Smith D . Mapping the cellular biogeography of human bone marrow niches using single-cell transcriptomics and proteomic imaging. Cell. (2024) 187:3120–3140 e3129. doi: 10.1016/j.cell.2024.04.013, PMID: 38714197 PMC11162340

[25] Jin S Guerrero-Juarez CF Zhang L Chang I Ramos R Kuan CH . Inference and analysis of cell-cell communication using CellChat. Nat Commun. (2021) 12:1088. doi: 10.1038/s41467-021-21246-9, PMID: 33597522 PMC7889871

[26] Ganesan N Ronsmans S Hoet P . Methods to assess proliferation of stimulated human lymphocytes. In Vitro: A Narrat Rev Cells. (2023) 12:386. doi: 10.3390/cells12030386, PMID: 36766728 PMC9913443

[27] Menard C Pacelli L Bassi G Dulong J Bifari F Bezier I . Clinical-grade mesenchymal stromal cells produced under various good manufacturing practice processes differ in their immunomodulatory properties: standardization of immune quality controls. Stem Cells Dev. (2013) 22:1789–801. doi: 10.1089/scd.2012.0594, PMID: 23339531 PMC3668498

[28] Ketterl N Brachtl G Schuh C Bieback K Schallmoser K Reinisch A . A robust potency assay highlights significant donor variation of human mesenchymal stem/progenitor cell immune modulatory capacity and extended radio-resistance. Stem Cell Res Ther. (2015) 6:236. doi: 10.1186/s13287-015-0233-8, PMID: 26620155 PMC4666276

[29] Roux C Saviane G Pini J Belaid N Dhib G Voha C . Immunosuppressive mesenchymal stromal cells derived from human-induced pluripotent stem cells induce human regulatory T cells. In Vitro In Vivo. Front Immunol. (2017), 8. doi: 10.3389/fimmu.2017.01991, PMID: 29422893 PMC5788894

[30] Nam Y Jung SM Rim YA Jung H Lee K Park N . Intraperitoneal infusion of mesenchymal stem cell attenuates severity of collagen antibody induced arthritis. PloS One. (2018) 13:e0198740. doi: 10.1371/journal.pone.0198740, PMID: 29879214 PMC5991665

[31] Negi N Griffin MD . Effects of mesenchymal stromal cells on regulatory T cells: Current understanding and clinical relevance. Stem Cells. (2020) 38:596–605. doi: 10.1002/stem.3151, PMID: 31995249 PMC7217190

[32] de Witte SFH Luk F Sierra Parraga JM Gargesha M Merino A Korevaar SS . Immunomodulation by therapeutic mesenchymal stromal cells (MSC) is triggered through phagocytosis of MSC by monocytic cells. Stem Cells. (2018) 36:602–15. doi: 10.1002/stem.2779, PMID: 29341339

[33] Lu D Xu Y Liu Q Zhang Q . Mesenchymal stem cell-macrophage crosstalk and maintenance of inflammatory microenvironment homeostasis. Front Cell Dev Biol. (2021) 9:681171. doi: 10.3389/fcell.2021.681171, PMID: 34249933 PMC8267370

[34] Li ZH Chen JF Zhang J Lei ZY Wu LL Meng SB . Mesenchymal stem cells promote polarization of M2 macrophages in mice with acute-on-chronic liver failure via mertk/JAK1/STAT6 signaling. Stem Cells. (2023) 41:1171–84. doi: 10.1093/stmcls/sxad069, PMID: 37659098

[35] Hashimoto S Yamada M Motoyoshi K Akagawa KS . Enhancement of macrophage colony-stimulating factor-induced growth and differentiation of human monocytes by interleukin-10. Blood. (1997) 89:315–21. doi: 10.1182/blood.V89.1.315, PMID: 8978307

[36] Delneste Y Charbonnier P Herbault N Magistrelli G Caron G Bonnefoy JY . Interferon-gamma switches monocyte differentiation from dendritic cells to macrophages. Blood. (2003) 101:143–50. doi: 10.1182/blood-2002-04-1164, PMID: 12393446

[37] Mia S Warnecke A Zhang XM Malmstrom V Harris RA . An optimized protocol for human M2 macrophages using M-CSF and IL-4/IL-10/TGF-beta yields a dominant immunosuppressive phenotype. Scand J Immunol. (2014) 79:305–14. doi: 10.1111/sji.12162, PMID: 24521472 PMC4282403

[38] Benner B Scarberry L Suarez-Kelly LP Duggan MC Campbell AR Smith E . Generation of monocyte-derived tumor-associated macrophages using tumor-conditioned media provides a novel method to study tumor-associated macrophages *in vitro*. J Immunother Cancer. (2019) 7:140. doi: 10.1186/s40425-019-0622-0, PMID: 31138333 PMC6540573

[39] Luque-Martin R Angell DC Kalxdorf M Bernard S Thompson W Eberl HC . IFN-gamma drives human monocyte differentiation into highly proinflammatory macrophages that resemble a phenotype relevant to psoriasis. J Immunol. (2021) 207:555–68. doi: 10.4049/jimmunol.2001310, PMID: 34233910

[40] Amorim A De Feo D Friebel E Ingelfinger F Anderfuhren CD Krishnarajah S . IFNgamma and GM-CSF control complementary differentiation programs in the monocyte-to-phagocyte transition during neuroinflammation. Nat Immunol. (2022) 23:217–28. doi: 10.1038/s41590-021-01117-7, PMID: 35102344

[41] Kadomoto S Izumi K Mizokami A . Macrophage polarity and disease control. Int J Mol Sci. (2021) 23:144. doi: 10.3390/ijms23010144, PMID: 35008577 PMC8745226

[42] Meisel R Zibert A Laryea M Gobel U Daubener W Dilloo D . Human bone marrow stromal cells inhibit allogeneic T-cell responses by indoleamine 2,3-dioxygenase-mediated tryptophan degradation. Blood. (2004) 103:4619–21. doi: 10.1182/blood-2003-11-3909, PMID: 15001472

[43] Francois M Copland IB Yuan S Romieu-Mourez R Waller EK Galipeau J . Cryopreserved mesenchymal stromal cells display impaired immunosuppressive properties as a result of heat-shock response and impaired interferon-gamma licensing. Cytotherapy. (2012) 14:147–52. doi: 10.3109/14653249.2011.623691, PMID: 22029655 PMC3279133

[44] Hansen SB Hojgaard LD Kastrup J Ekblond A Follin B Juhl M . Optimizing an immunomodulatory potency assay for Mesenchymal Stromal Cell. Front Immunol. (2022) 13:1085312. doi: 10.3389/fimmu.2022.1085312, PMID: 36578497 PMC9791065

[45] Pallotta MT Rossini S Suvieri C Coletti A Orabona C Macchiarulo A . Indoleamine 2,3-dioxygenase 1 (IDO1): an up-to-date overview of an eclectic immunoregulatory enzyme. FEBS J. (2022) 289:6099–118. doi: 10.1111/febs.16086, PMID: 34145969 PMC9786828

[46] Munn DH Shafizadeh E Attwood JT Bondarev I Pashine A Mellor AL . Inhibition of T cell proliferation by macrophage tryptophan catabolism. J Exp Med. (1999) 189:1363–72. doi: 10.1084/jem.189.9.1363, PMID: 10224276 PMC2193062

[47] Qin R Zhao C Wang CJ Xu W Zhao JY Lin Y . Tryptophan potentiates CD8(+) T cells against cancer cells by TRIP12 tryptophanylation and surface PD-1 downregulation. J Immunother Cancer. (2021) 9:e002840. doi: 10.1136/jitc-2021-002840, PMID: 34326168 PMC8323461

[48] Hess NJ Brown ME Capitini CM . GVHD pathogenesis, prevention and treatment: lessons from humanized mouse transplant models. Front Immunol. (2021) 12:723544. doi: 10.3389/fimmu.2021.723544, PMID: 34394131 PMC8358790

[49] Kowal K Silver R Slawinska E Bielecki M Chyczewski L Kowal-Bielecka O . CD163 and its role in inflammation. Folia Histochem Cytobiol. (2011) 49:365–74. doi: 10.5603/FHC.2011.0052, PMID: 22038213

[50] Hu JM Liu K Liu JH Jiang XL Wang XL Chen YZ . CD163 as a marker of M2 macrophage, contribute to predicte aggressiveness and prognosis of Kazakh esophageal squamous cell carcinoma. Oncotarget. (2017) 8:21526–38. doi: 10.18632/oncotarget.15630, PMID: 28423526 PMC5400603

[51] Branchett WJ Saraiva M O’Garra A . Regulation of inflammation by Interleukin-10 in the intestinal and respiratory mucosa. Curr Opin Immunol. (2024) 91:102495. doi: 10.1016/j.coi.2024.102495, PMID: 39357078

[52] Giri J Das R Nylen E Chinnadurai R Galipeau J . CCL2 and CXCL12 derived from mesenchymal stromal cells cooperatively polarize IL-10+ Tissue macrophages to mitigate gut injury. Cell Rep. (2020) 30:1923–1934 e1924. doi: 10.1016/j.celrep.2020.01.047, PMID: 32049021 PMC7043065

[53] Whelan DS Caplice NM Clover AJP . Mesenchymal stromal cell derived CCL2 is required for accelerated wound healing. Sci Rep. (2020) 10:2642. doi: 10.1038/s41598-020-59174-1, PMID: 32060374 PMC7021763

[54] Miao L Yu C Guan G Luan X Jin X Pan M . Extracellular vesicles containing GAS6 protect the liver from ischemia-reperfusion injury by enhancing macrophage efferocytosis via MerTK-ERK-COX2 signaling. Cell Death Discov. (2024) 10:401. doi: 10.1038/s41420-024-02169-y, PMID: 39256347 PMC11387478

[55] Sheta M Taha EA Lu Y Eguchi T . Extracellular vesicles: new classification and tumor immunosuppression. Biol (Basel). (2023) 12:110. doi: 10.3390/biology12010110, PMID: 36671802 PMC9856004

[56] Garcia-Contreras M Thakor AS . Author Correction: Human adipose tissue-derived mesenchymal stem cells and their extracellular vesicles modulate lipopolysaccharide activated human microglia. Cell Death Discov. (2024) 10:449. doi: 10.1038/s41420-024-02209-7, PMID: 39443449 PMC11500170

[57] de Couto G Jaghatspanyan E DeBerge M Liu W Luther K Wang Y . Mechanism of enhanced merTK-dependent macrophage efferocytosis by extracellular vesicles. Arterioscler Thromb Vasc Biol. (2019) 39:2082–96. doi: 10.1161/ATVBAHA.119.313115, PMID: 31434491 PMC6760997

[58] Tkach M Thalmensi J Timperi E Gueguen P Nevo N Grisard E . Extracellular vesicles from triple negative breast cancer promote pro-inflammatory macrophages associated with better clinical outcome. Proc Natl Acad Sci U.S.A. (2022) 119:e2107394119. doi: 10.1073/pnas.2107394119, PMID: 35439048 PMC9169908

[59] Bournazos S Wang TT Ravetch JV . The role and function of fcgamma receptors on myeloid cells. Microbiol Spectr. (2016) 4:10. doi: 10.1128/microbiolspec.MCHD-0045-2016, PMID: 28087938 PMC5240797

[60] Isenberg JS Roberts DD . THBS1 (thrombospondin-1). Atlas Genet Cytogenet Oncol Haematol. (2020) 24:291–9. doi: 10.4267/2042/70774, PMID: 33244322 PMC7687907

[61] Tossetta G Piani F Borghi C Marzioni D . Role of CD93 in health and disease. Cells. (2023) 12:1778. doi: 10.3390/cells12131778, PMID: 37443812 PMC10340406

[62] Raoufi A Soleimani Samarkhazan H Nouri S Khaksari MN Abbasi Sourki P Sargazi Aval O . Macrophages in graft-versus-host disease (GVHD): dual roles as therapeutic tools and targets. Clin Exp Med. (2025) 25:73. doi: 10.1007/s10238-025-01588-0, PMID: 40048037 PMC11885342

[63] Halder SK Beauchamp RD Datta PK . A specific inhibitor of TGF-beta receptor kinase, SB-431542, as a potent antitumor agent for human cancers. Neoplasia. (2005) 7:509–21. doi: 10.1593/neo.04640, PMID: 15967103 PMC1501161

[64] Fleischauer J Bastone AL Selich A John-Neek P Weisskoeppel L Schaudien D . TGFbeta inhibitor A83–01 enhances murine HSPC expansion for gene therapy. Cells. (2023) 12:509–21. doi: 10.1073/pnas.1401343111, PMID: 37566057 PMC10416825

[65] Ferraro A D’Alise AM Raj T Asinovski N Phillips R Ergun A . Interindividual variation in human T regulatory cells. Proc Natl Acad Sci U.S.A. (2014) 111:E1111–1120. doi: 10.1073/pnas.1401343111, PMID: 24610777 PMC3970507

[66] Su LF Del Alcazar D Stelekati E Wherry EJ Davis MM . Antigen exposure shapes the ratio between antigen-specific Tregs and conventional T cells in human peripheral blood. Proc Natl Acad Sci U.S.A. (2016) 113:E6192–8. doi: 10.1073/pnas.1611723113, PMID: 27681619 PMC5068288

[67] Pittenger MF Discher DE Peault BM Phinney DG Hare JM Caplan AI . Mesenchymal stem cell perspective: cell biology to clinical progress. NPJ Regener Med. (2019) 4:22. doi: 10.1038/s41536-019-0083-6, PMID: 31815001 PMC6889290

[68] Malicev E Jazbec K . An overview of mesenchymal stem cell heterogeneity and concentration. Pharm (Basel). (2024) 17:22. doi: 10.3390/ph17030350, PMID: 38543135 PMC10975472

[69] Teo KYW Zhang S Loh JT Lai RC Hey HWD Lam KP . Mesenchymal stromal cell exosomes mediate M2-like macrophage polarization through CD73/ecto-5’-nucleotidase activity. Pharmaceutics. (2023) 15:1550280. doi: 10.3390/pharmaceutics15051489, PMID: 37242732 PMC10220822

[70] Fallah A Hosseinzadeh Colagar A Khosravi A Mohammad-Hasani A Saeidi M . The role of natural exosomes from SHED-MSC in immunoregulation of M0/M1 polarized macrophage cells. Front Immunol. (2025) 16:1550280. doi: 10.3389/fimmu.2025.1550280, PMID: 39991155 PMC11842380

[71] Lee RH Pulin AA Seo MJ Kota DJ Ylostalo J Larson BL . Intravenous hMSCs improve myocardial infarction in mice because cells embolized in lung are activated to secrete the anti-inflammatory protein TSG-6. Cell Stem Cell. (2009) 5:54–63. doi: 10.1016/j.stem.2009.05.003, PMID: 19570514 PMC4154377

[72] Eggenhofer E Benseler V Kroemer A Popp FC Geissler EK Schlitt HJ . Mesenchymal stem cells are short-lived and do not migrate beyond the lungs after intravenous infusion. Front Immunol. (2012) 3:297. doi: 10.3389/fimmu.2012.00297, PMID: 23056000 PMC3458305

[73] Dave M Dev A Somoza RA Zhao N Viswanath S Mina PR . MSCs mediate long-term efficacy in a Crohn’s disease model by sustained anti-inflammatory macrophage programming via efferocytosis. NPJ Regener Med. (2024) 9:6. doi: 10.1038/s41536-024-00347-1, PMID: 38245543 PMC10799947

[74] Li A Guo F Pan Q Chen S Chen J Liu HF . Mesenchymal stem cell therapy: hope for patients with systemic lupus erythematosus. Front Immunol. (2021) 12:728190. doi: 10.3389/fimmu.2021.728190, PMID: 34659214 PMC8516390

[75] Hori S . FOXP3 as a master regulator of T(reg) cells. Nat Rev Immunol. (2021) 21:618–9. doi: 10.1038/s41577-021-00598-9, PMID: 34580451

[76] FDA . Guidance for industry: potency tests for cellular and gene therapy products (2011). Available online at: https://www.fda.gov/files/vaccines,%20blood%20%26%20biologics/published/Final-Guidance-for-Industry--Potency-Tests-for-Cellular-and-Gene-Therapy-Products.pdf (Accessed November 19, 2025).

