# Supplementary figures and images for: Development and validation of a potency assay matrix for optimized and consistent manufacture of clinical mesenchymal stem/stromal cells
Source: Front Immunol. 2026 Feb 19;17:1725191. doi: 10.3389/fimmu.2026.1725191 (PMC12960169; doi:10.3389/fimmu.2026.1725191)

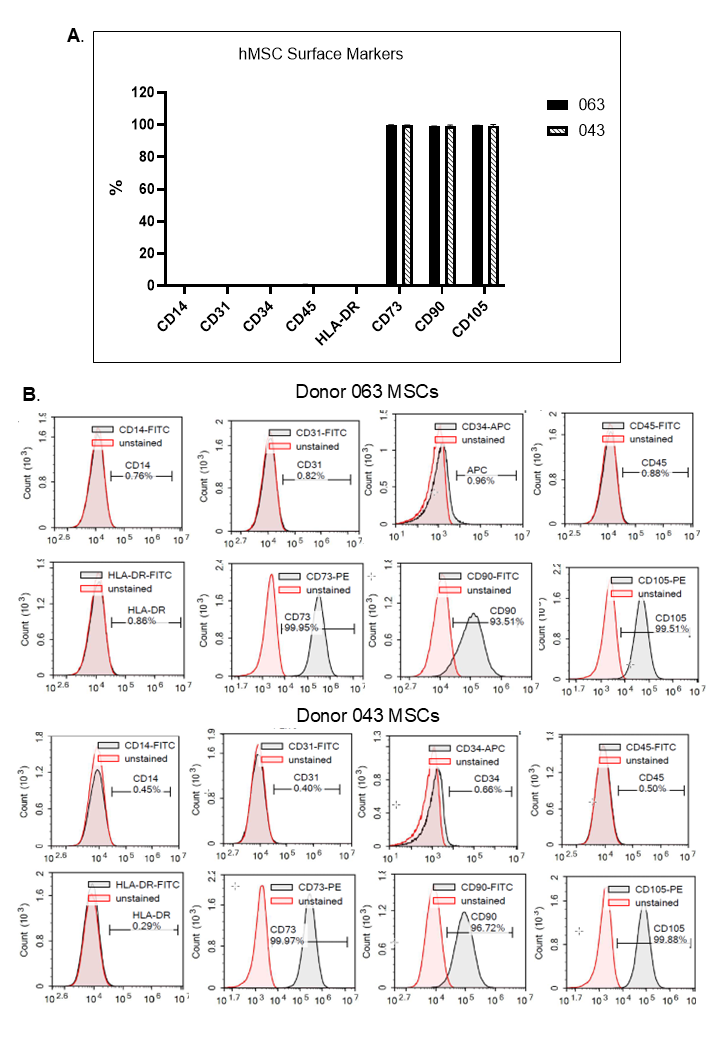

Supplement: Supplementary Figure 1 — Characterization of MSCs. MSCs from donors 043 and 063 were cultured to passage 4 to assess surface marker phenotype, colony forming unit-fibroblast (CFU-F) potential and trilineage differentiation. (A) Means ± SD for expression of each surface marker. (B) Representative flow cytometry histograms. (C) Means ± SD for 14 day CFU-F assays. (D) Representative images of MSCs after adipogenic, osteogenic and chondrogenic differentiation and staining. [file Image1.tif]

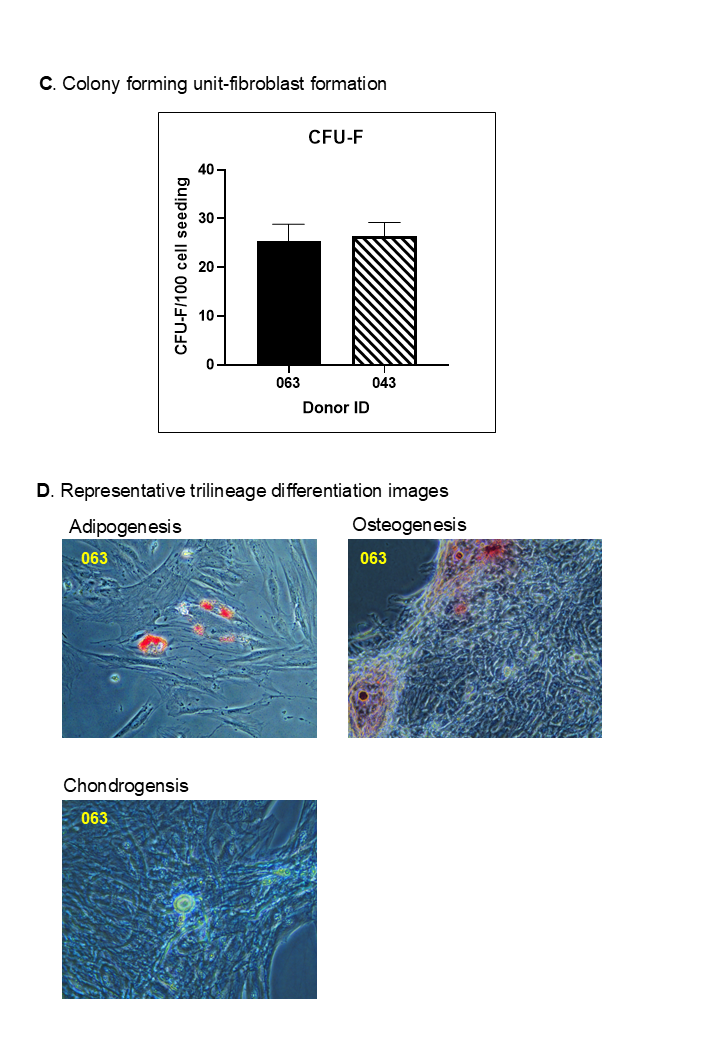

Supplement: Supplementary Figure 2 — MSCs suppress proliferation of isolated T cells. (A) Representative flow cytometry plots and quantification of CFSE dilution and replication index of CD4+ T cells cultured alone (No MSCs) or co-cultured with 20,000 (20k) or 40,000 (40k) MSCs (MSC donor 063). (B) Representative flow cytometry plots and quantification of CFSE dilution and replication index of CD8+ T cells cultured alone (No MSCs) or co-cultured with 20,000 (20k) or 40,000 (40k) MSCs (MSC donor 063). Data are means ± SD. n ≥ 3 technical replicates using one PBMC donor. Statistical significance was determined using one-way ANOVA with Tukey’s HSD test. **P ≤ 0.01; ****P ≤ 0.0001. [file Image2.tif]

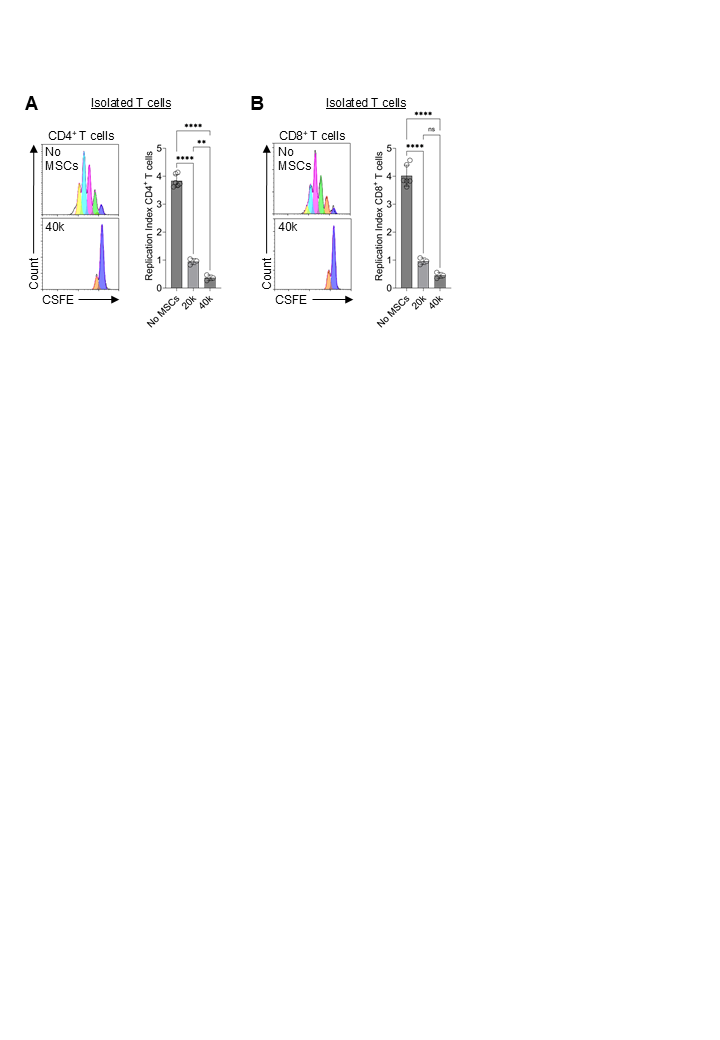

Supplement: Supplementary Figure 3 — Cluster heatmap of monocytes differentiated to M1 or M2 macrophages or treated with MSC-CM. (A) Schematic presentation of bulk RNA-seq of monocytes. (B) Cluster heatmap showing DEGs with a Log2 fold change of ≥ 3. (C) Cluster heatmap of DEGs with a Log2 fold change of ≥ 1 plus zoom-in showing genes of selected clusters. [file Image3.tif]

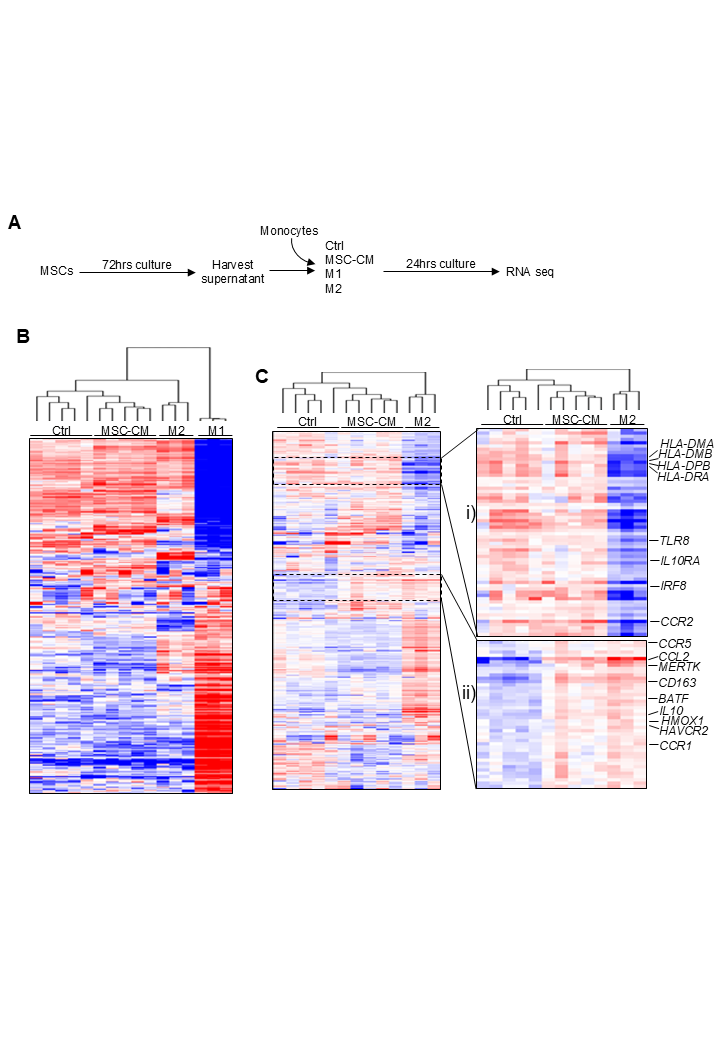

Supplement: Supplementary Figure 4 — Quantification of MSC-derived EVs. (A) Western blot analysis of EVs and 063 MSC whole cell lysates. Each lane contained 15 µg total protein. Molecular weights were estimated based on MW size markers. (B) Size distribution of MSC-derived EVs measured by NTA. (C) Average size of EVs. (D) Average number of EVs/mL. Data are means ± SD. n = 3 independent MSC-EV preparations. (E) Correlation between CD63 levels measured via CD63 ELISA and EV numbers measured by NTA. (F) Representative flow images of EVs isolated with CD63 capture beads and stained for CD63 and CD73. [file Image4.tif]

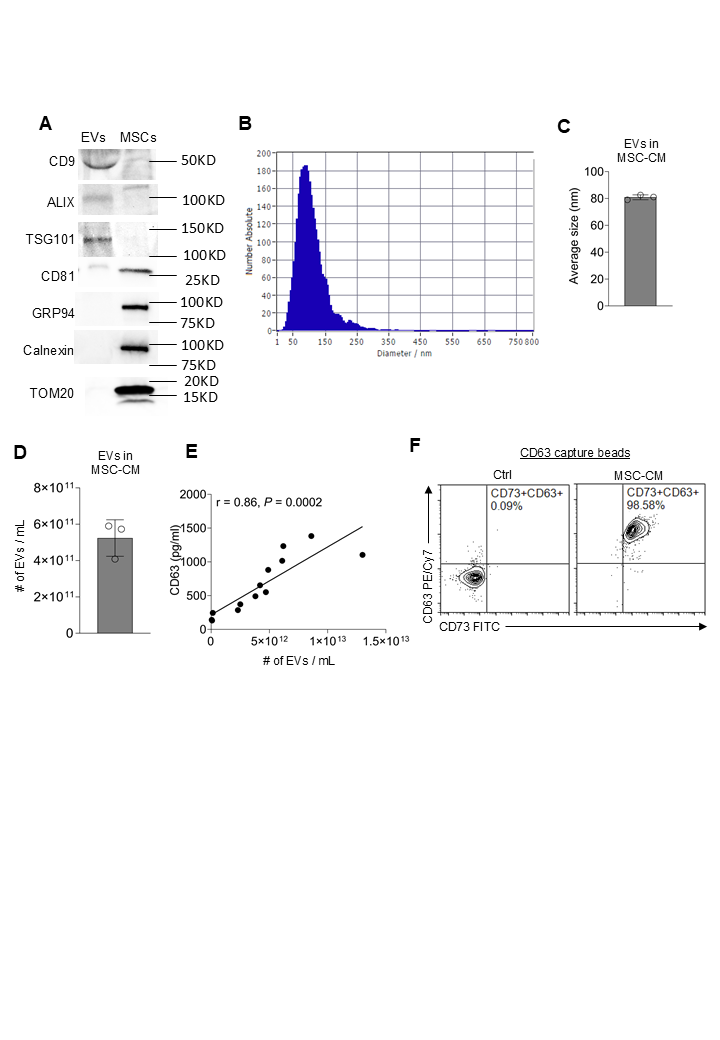

Supplement: Supplementary Figure 5 — MerTK- and M-CSF-signaling mediate EV-driven M2 polarization. (A) Representative flow images of live CD45+ monocytes incubated with CBO-labeled EVs derived from MSCs or unlabeled EVs. (B) Representative flow image of CD163 expression in monocytes treated with CBO-labeled EVs (top) and MFI of CD163 in CBO- vs CBO+ monocytes from the same sample (bottom). Data are means ± SD. n = 4 independent experiments using four different PBMC donors. Statistical significance was determined using a paired t-test. [file Image5.tif]

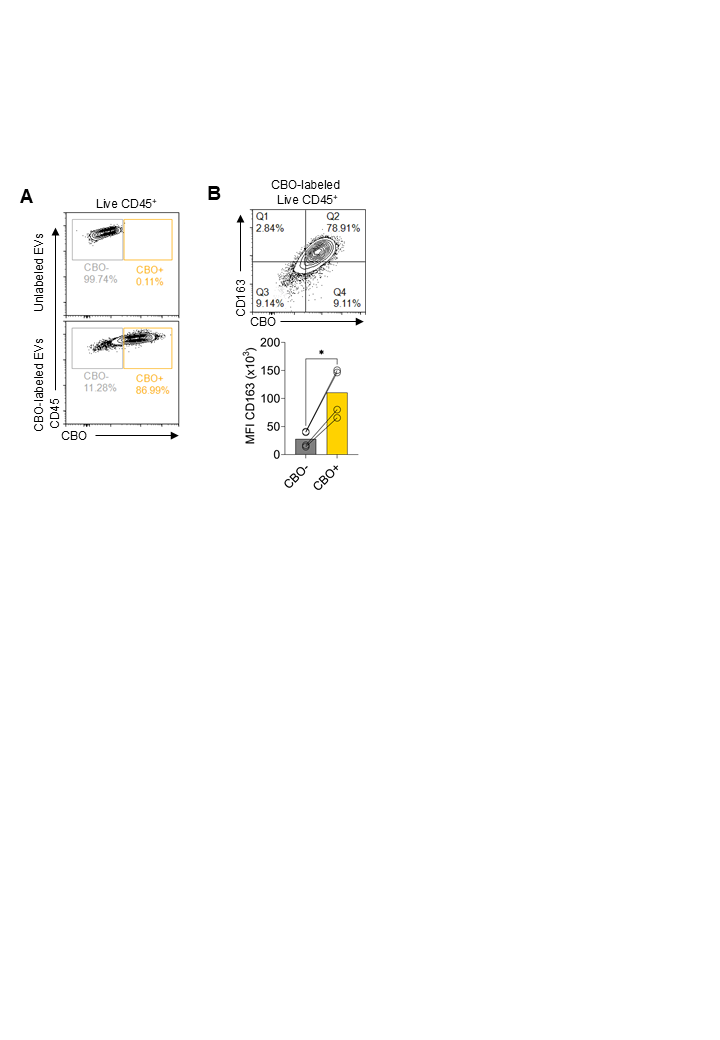

Supplement: Supplementary Figure 6 — Extracellular vesicles released by MSCs increase phagocytic capacity of macrophages. (A) Representative imaging flow cytometry images of IgG-FITC opsonized microbeads added to monocytes differentiated to M1 or M2 macrophages, monocytes primed with MSCs-EVs or EVs isolated from cRPMI (B) Quantification of IgG-FITC microbeads uptake as described in (A). (C) Representative flow images of IgG-FITC-positive monocytes. Data are means ± SD. n = 2 independent experiments using two different PBMC donors. [file Image6.tif]

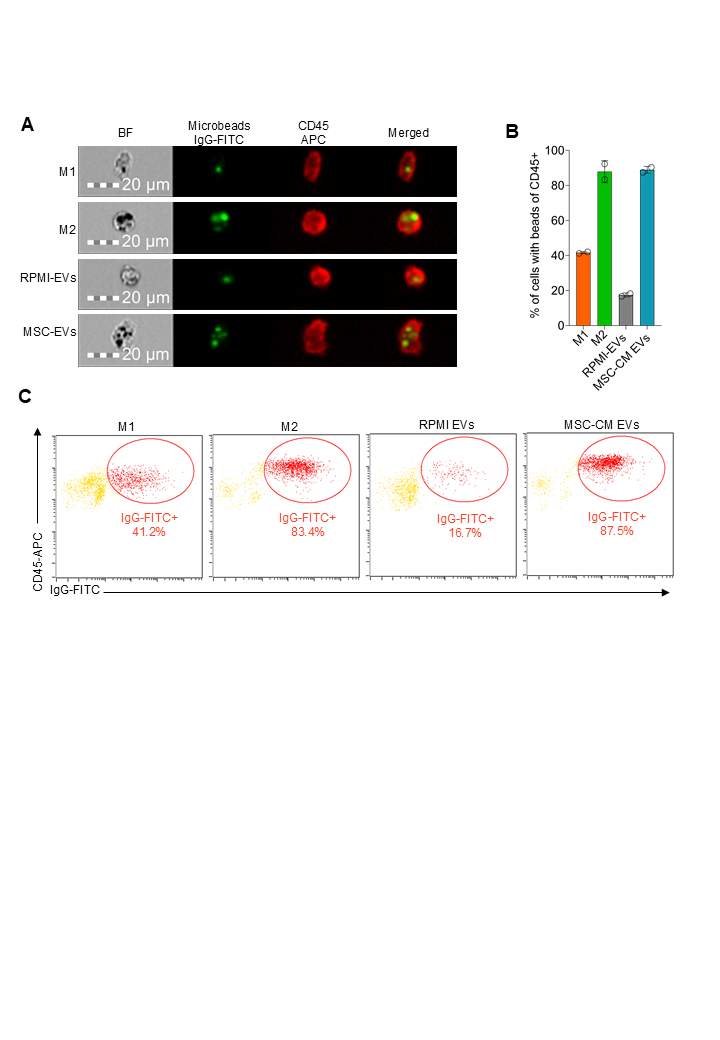

Supplement: Supplementary Figure 7 — Active TGFβ1 on EVs surface drives Treg differentiation. (A) Schematic overview of the experiment. MSC-derived EVs were incubated for 3hrs at 37 °C with an anti-TGFβ1 antibody or isotype control and either added directly to T cells (No Wash) or unbound antibodies were washed away, and EVs were re-isolated (Wash). (B) Frequency of FoxP3+ among CD4+ T cells stimulated with αCD3/αCD28 beads + IL-2 and cultured as described in (A). Data are means ± SD. n = 3 technical replicates using one PBMC donor. (C) Frequency of FoxP3+ among CD4+ T cells stimulated with αCD3/αCD28 beads + IL-2 and culture with EVs, EVs + anti-LAP antibody, EVs + isotype control or without EVs (Ctrl). Data are means ± SD. N = 3 technical replicates using one PBMC donor. (D) TGFβ1 concentrations on EVs measured via TGFβ1 ELISA without HCl activation of samples. Additionally, the EVs were incubated with 13 µg/mL anti-LAP antibody or isotype control. Data are means ± SD. n = 3 technical replicates. Statistical significance was determined using a one-way ANOVA with Tukey’s HSD test. *P ≤ 0.05, **P ≤ 0.01. [file Image7.tif]

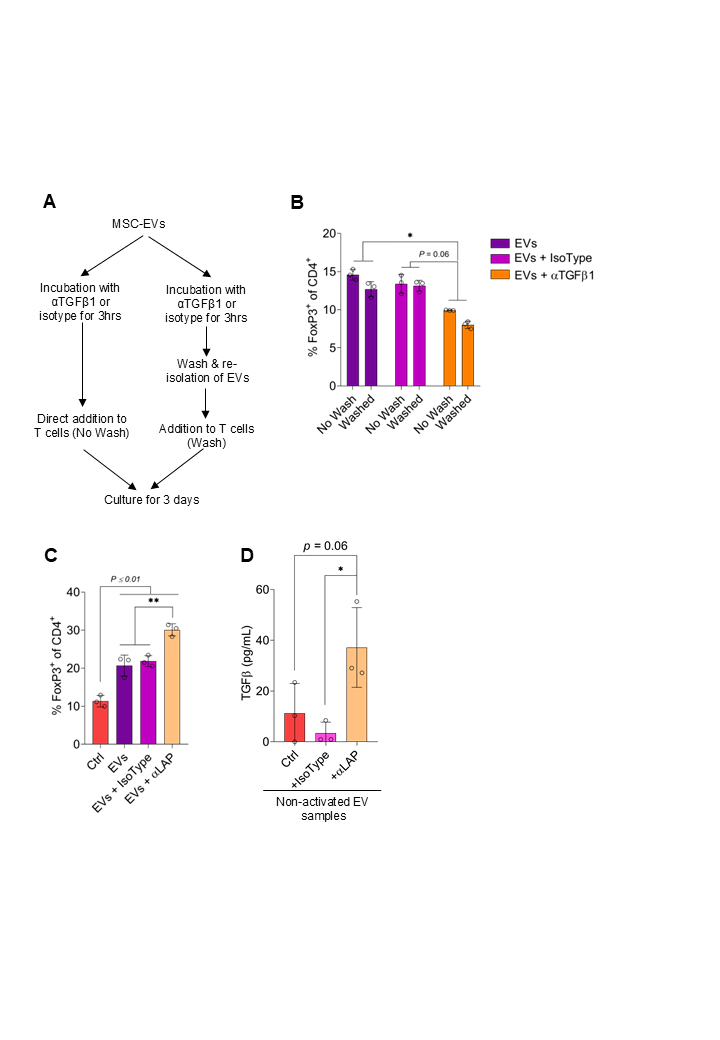

Supplement: Supplementary Figure 8 — Representative flow cytometry gating strategy. Example of the gating strategies for (A) CD4+ and CD8+ CSFE labeled stimulated T cells, (B) FoxP3 expression in the CD4+ T cell population, (C) IDO expression by MSCs, (D) monocytes/macrophages in PBMCs, and (E) Determining monocyte migration [file Image8.tif]

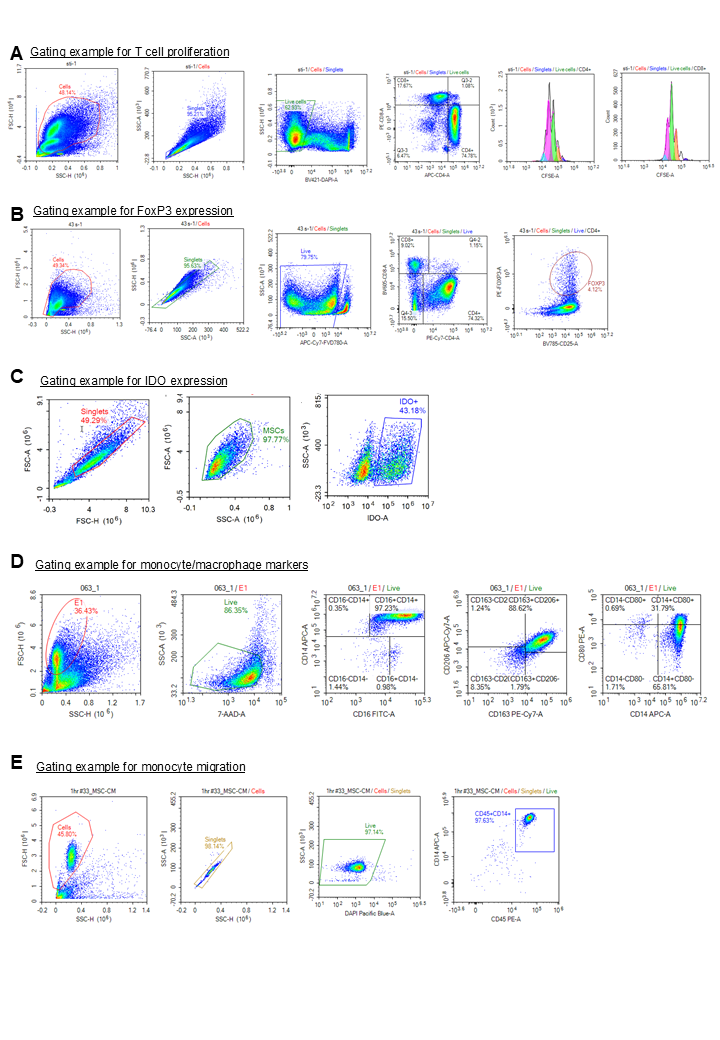

Supplement: Supplementary file 9 [file Image9.tif]
